# Multiple Kernel Learning Model for Relating Structural and Functional Connectivity in the Brain

**DOI:** 10.1038/s41598-018-21456-0

**Published:** 2018-02-19

**Authors:** Sriniwas Govinda Surampudi, Shruti Naik, Raju Bapi Surampudi, Viktor K. Jirsa, Avinash Sharma, Dipanjan Roy

**Affiliations:** 1 CVIT, IIIT-Hyderabad, Hyderabad, 500032 India; 2 Cognitive Science Lab, IIIT-Hyderabad, Hyderabad, 500032 India; 3 School of Computer and Information Sciences, University of Hyderabad, Hyderabad, 500046 India; 4 Aix Marseille Univ, Inserm, INS, Institut de Neurosciences des Systèmes, Marseille, France; 5 Cognitive Brain Dynamics Lab, National Brain Research Centre, Manesar, Gurgaon, Haryana 122 051 India

## Abstract

A challenging problem in cognitive neuroscience is to relate the structural connectivity (SC) to the functional connectivity (FC) to better understand how large-scale network dynamics underlying human cognition emerges from the relatively fixed SC architecture. Recent modeling attempts point to the possibility of a single diffusion kernel giving a good estimate of the FC. We highlight the shortcomings of the single-diffusion-kernel model (SDK) and propose a multi-scale diffusion scheme. Our multi-scale model is formulated as a reaction-diffusion system giving rise to spatio-temporal patterns on a fixed topology. We hypothesize the presence of inter-regional co-activations (latent parameters) that combine diffusion kernels at multiple scales to characterize how FC could arise from SC. We formulated a multiple kernel learning (MKL) scheme to estimate the latent parameters from training data. Our model is analytically tractable and complex enough to capture the details of the underlying biological phenomena. The parameters learned by the MKL model lead to highly accurate predictions of subject-specific FCs from test datasets at a rate of 71%, surpassing the performance of the existing linear and non-linear models. We provide an example of how these latent parameters could be used to characterize age-specific reorganization in the brain structure and function.

## Introduction

Over the last two decades, investigation of the slow correlated fluctuations in the resting state functional magnetic resonance images (rs-fMRI) of the brain have yielded valuable insights about the spontaneous functional organization of the brain. The structural connectivity (SC) derived from diffusion tensor imaging (DTI) reveals the white-matter fiber connections between regions of interest (ROIs)^1,2^. On the other hand, functional connectivity (FC) estimates the linear statistical dependency between ROIs based on blood oxygen level dependent (BOLD) activation^3^. It has been observed that BOLD signals are non-stationary in nature^4–6^. This non-stationarity is understood in terms of states (measured in small time windows) and their transitions and are expressed as functional connectivity dynamics (FCD)^7^. On the other hand, static FC enables investigation of the analytical properties of network behavior^8^.

Even in resting conditions BOLD activations of ROIs have been observed to form self-organizing patterns called resting state networks (RSNs). FC can be interpreted as a superposition of all these RSNs. Relating structural connectivity (SC) of the brain to its functional connectivity (FC) is a fundamental goal in neuroscience because it enables our understanding of how the relatively fixed SC architecture underlies human cognition and diverse behaviors^9^. It has been observed that regions with direct structural link are also functionally connected. However the converse is not necessarily true^10^. The question of how SC shapes FC has been the object of computational modeling but remains an open question^11^. In the recent years, connectivity analysis using whole brain computational models and graph theoretic techniques have given unprecedented insights about brain-wide correlations during rest and task conditions^12–14^. Computational models are designed to expand our understanding and explaining the functioning of the brain. The more biologically real the model is, the more computationally expensive it is. Hence, gaining analytical insights becomes increasingly difficult with complex models.

In the realm of noise-induced correlated deviations, there are linear and non-linear mean field models that attempt to answer this open question incorporating various kinds of dynamics and biological details^15–18^. A biophysical attempt to relate SC to FC is a linear model based on graph diffusion of brain dynamics is outlined in^18^. This linear diffusion model considers brain dynamics, the diffusing quantity, firing rate of the neuronal population, undergoing random walk on the SC graph. This linear diffusion model considers that the mean regional activity diffuses over the anatomical fibers governed by a deterministic linear differential equation^18^. The analytically tractable solution becomes the graph diffusion kernel which is hypothesized to resemble the FC. This model fixes one global parameter across all subjects. Another model proposed by Saggio *et al*.^19^ considers a linear auto-regressive model with additive Gaussian white noise, coupling matrix being SC. This model becomes a linear system of coupled stochastic first order differential equations in which the BOLD activities diffuse on the anatomical constraints, i.e. SC. This model computes covariance between regional activities whose analytical expression works out to be a function of SC. Such a model would find it difficult to account for inter-subject variability in the functional expression. Extending the idea of linearity to super-critical bifurcations and multi-stability, a series of non-linear stochastic models have been proposed that explain the underlying biological behavior efficiently^20–22^. These models differ in their representation scheme for the ROIs^23^. Whereas Kuramoto oscillator model^24^ abstracts out the biophysical details, Deco and Jirsa’s mean-field-models^20,21^ consider dynamics of specific biological analogues such as mean firing rate and mean activity of the regions. These neural and meso-scopic models can be seen as variants of reaction-diffusion system at the heart of which lie the Wilson-Cowan equations^25,26^. Wilson-Cowan equations, a variant of reaction-diffusion systems, provide a coarse-grained description of the large-scale neuronal network in terms of oscillatory self-organizing patterns. New experimental evidence supports these equations^27^.

Recently, a new paradigm of understanding the oscillatory patterns of cortico-cortical activity is proposed that utilizes spectral analysis of the connectome or structural connectivity (SC)^28^. It has been observed that these connectome-specific harmonics predict oscillatory functional networks of the human brain possibly through interplay of excitation; for instance mediated by the glutamatergic principal cells, and inhibition; for instance mediated by the GABAergic interneurons. The push-and-pull between diffusing excitatory cells and suppressing inhibitory cells can result in self-organizing pattern formation. The emergent harmonics or the standing waves are the allowed spatial frequencies, or the eigenfunctions of the graph Laplacian operator on the anatomically constrained SC largely determined by the selection of the diffusion parameters of excitation and inhibition. Surampudi *et al*.^29^ observed that physical diffusion on large-scale graphs, i.e. SC, at multiple *diffusion scales* exhibits scale-dependent relationships among various regions of interest (ROIs). These multi-scale diffusion kernels are similarly motivated to capture reaction-diffusion systems operating on a fixed underlying connectome (SC) and hence can be interpreted as components of FC at different diffusion scales (see Supplementary Fig. S1 for the motivation of multi-scale approach). However, our investigations revealed that a combination of multiple diffusion kernels was not sufficient to explain the self-organizing resting state patterns found in FC and hence necessitates the need of additional explanatory parameters.

The extant whole brain computational models can be characterized along two dimensions–interpretability and complexity, where the linear and non-linear models lie at the opposite ends of the spectrum. The former are analytical models with few parameters that can be interpreted and tuned easily, whereas the latter are fairly complex models with richer dynamics but tend to be analytically intractable. The proposed model possesses the analytical beauty of linear models and yet is complex enough to capture the biological details. We hypothesize that the presence of regional multi-scale co-activations that initiate diffusion would be necessary to bridge the gap between structurally confined diffusion phenomenon and empirically observed FC and that these co-activations would be common across the cohort. We further provide a plausible mathematical reasoning for the existence of these co-activations along with diffusion kernels by linearizing a variant of reaction-diffusion model and extending it to generate FC. Moreover, we also describe a succinct multiple kernel learning (MKL) procedure to retrieve these co-activations by formulating it as an optimization formulation. MKL techniques are well explored in the machine learning community^30,31^. Our proposed model while retaining the parsimony of a simple linear approach, proposes a novel learning scheme for optimizing the best-fitting kernels for SC-to-FC mapping. Our detailed empirical results demonstrate the validity of the proposed model on a larger dataset.

## Results

### Model description

Our model is based on the hypothesis that there are inter-regional multi-scale co-activations (among ROIs) for initiating diffusion. These co-activations that are shared across all subjects enable our model to generate the observed FC patterns from the structurally confined diffusion kernels. This is achieved by first post-multiplying the co-activation parameters (denoted henceforth as *π*_*i*_′s) with the respective diffusion kernel matrices derived from subject-specific structural matrix (SC) and then taking a linear combination of these for approximating/predicting the functional connectivity (FC). We propose to learn these co-activation parameters in a multiple kernel learning (MKL) framework, thus retaining the parsimony of a simple linear approach. In the experiments reported in this paper, 16 diffusion kernels were used and their respective inter-regional co-activation parameters (16 corresponding *π*_*i*_′s) were learned (see Supplementary Figs S2–S4 along with their corresponding sections for scale selection methodology). To evaluate our model, we mainly used the data scanned at the Berlin Centre for Advanced Imaging, Charité University, Berlin, Germany that has SC-FC pairs of total 47 subjects (see Supplementary section 8 for data pre-processing methodology).

### Model performance

We compared performance of the proposed model (henceforth designated as MKL model) with two previously proposed approaches: single-diffusion-kernel (SDK) model of Abdelnour *et al*.^18^ and the non-linear dynamic-mean-field (DMF) model described in Deco *et al*.^32^ (see *Methods* for implementation details of these models). To remain consistent with the previous studies, we used Pearson correlation coefficient between empirical and predicted functional connectivities (FC) as the measure of model performance. To obtain a benchmark, we computed the Pearson correlation between empirical SC-FC pairs for all subjects and found mean value for these correlations to be 0.3 with a standard deviation of 0.02. These values are taken as baseline correlation values henceforth.

**Figure 1:**
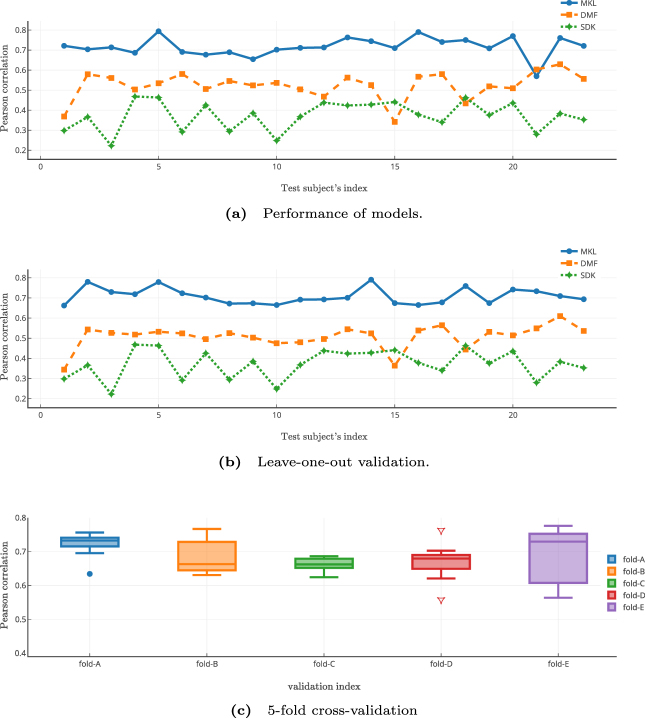
Comparison of Model Performance on Individual Test subjects. (**a**) Pearson correlation between empirical and predicted FCs of all the test subjects by multiple kernel learning (MKL) model and performance comparison with the predictions by the other two models. While MKL model has superior performance compared to that of dynamic mean field (DMF) and single diffusion kernel (SDK), DMF model performs slightly better than the SDK model. (**b**) Results of leave-one-out cross-validation on the test subjects also yield similar comparative performance. Note that the subject indices are kept identical between sub-figures (a) and (b). This plot suggests that MKL model can handle an increase in the number of training subjects without necessarily any over-fitting. (**c**) Box-plots of Pearson correlation measure on 9 randomly chosen validation subjects for each of the 5 folds for the MKL model. Points lying outside the quartiles are the suspected outliers. Compactness of boxes suggests inter-subject consistency of model’s performance. Further, these 5-fold cross-validation results suggest that MKL model performs consistently well on unseen subjects.

Figure 1 shows the performance comparison of the proposed method with other two models in three different setups. In the first setup, a randomly chosen set of half of the subjects was used for training (23 pairs) and the other half (23 pairs) for testing. Figure 1(a) shows the model performance for all the test participants for the three models. Since SDK and DMF models do not incorporate learning in their formulation, we gleaned the optimal values based on the training subjects. The optimal parameter settings were taken as the values at the mode of the performance distribution histogram for the training set and the same were used for estimating FC for test subjects. We took the best fitting scale on the training subjects for the SDK model and similarly selected the optimal global coupling parameter, *G*, for the DMF model. Optimal scale for SDK model worked out to be 0.8 and similarly optimal value of *G* for DMF was 2.85. As can be seen from Fig. 1(a) and (b), the MKL model performs consistently better for each test subject when compared to the other two models. In the remaining two setups, in order to crosscheck whether MKL model suffers from over-fitting, we computed leave-one-out (Fig. 1b) and 5-fold cross-validation (Fig. 1(c)) results. These results clearly show the consistency in the performance of MKL model and indicate that the performance is not due to over-fitting nor it is due to any particular optimistic train-test split.

**Figure 2:**
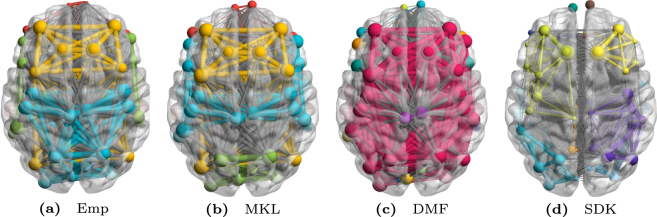
Functional Connectivity (FC) Networks Derived from Group-Mean FCs of the Test Dataset. The mean FC networks depict edge-connectivity patterns for (**a**) Empirical FC and FCs predicted by (**b**) MKL model; (**c**) DMF model^32^ and (**d**) SDK model^18^, respectively. Note the similarity of the MKL model and the empirical FC in terms of community assignment and inter-hemispheric connections. DMF model predicts a denser network while the single scale model predicts coarser network than the empirically observed FC. Brain-net-viewer^47^ was used for visualization of the four communities detected from the Louvian algorithm available in brain-connectivity-toolbox^48^. Colors of the edges and nodes are only to demarcate the communities observed and do not have any correspondence across brain surfaces for different models. Thus the comparison of community structure across models is qualitative in nature.

In all the experiments and for all the three models in order to compare group statistics, we compute the predicted FC for each test subject and then find the Pearson correlation coefficient with the corresponding empirical FC, followed by taking the mean of all these correlation coefficients. We designate the resulting mean correlation as mean FC in the rest of the paper.

### Edge-Connectivity Analysis

Mean FCs are visualized primarily in two modes, via the edge-connectivity pattern analysis and using the seed-based connectivity analysis. To understand the edge and node distribution across the communities, we rendered the mean predicted FCs on brain surface. The visualization of edge-connectivity patterns of four mean FCs is shown in Fig. 2 (see Supplementary Fig. S6 for the estimated FCs). In the figure, the colors demarcate the communities for a particular model on the corresponding brain surface. It can be seen that the community structure of the mean FC predicted by the MKL model (shown in Fig. 2(b)) best resembles that of the mean empirical FC (shown in Fig. 2(a)). The other two models predict either a dense FC network (as shown in Fig. 2(c) for the DMF model) or a sparse FC network (as shown in Fig. 2(d) for the SDK model), where both the scenarios are far from the empirically observed network. Additionally, the predicted mean FC by MKL model and the empirical mean FC seem similar in terms of community assignment and inter-hemispheric connections.

**Figure 3:**
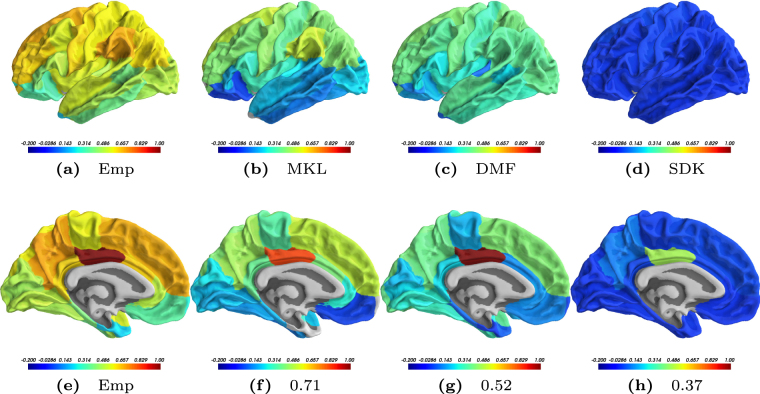
Results of Seed-based Correlation. Mean correlation maps resulting from considering the left Posterior Cingulate Cortex as a seed region and then calculating the seed-based correlations of all other regions. These maps are rendered on the left lateral sagittal view in the top sub-figures (**a**–**d**) and on the medial sagittal surface in the bottom sub-figures (**e**–**h**). While sub-figures (**a**) and (**e**) depict the maps for Empirical FC, the maps from the predicted FCs of MKL model are in (**b**) and (**f**); those of DMF in (**c**) and (**g**); and those of SDK in (**d**) and (**h**), respectively. Captions in the top row mention the model name and those in the bottom row indicate the mean correlation value on the test subjects. As can be observed, the correlation maps of MKL model seem to have greater correspondence with those of the mean empirical FC. Moreover, as depicted by the contrasts in the colors, MKL model is able to distinguish between the correlations at a better resolution than the other two models.

Further, to see element-wise variance in the mean predicted FCs, we also drew scatter plots between the predicted and empirical FCs in Supplementary Fig. S7, where only the non-diagonal lower triangular matrix entries were displayed. These plots suggest that MKL model preserves the global structure of the empirical FC as well as the element-wise connectivity patterns significantly better than the other models.

### Seed-based Connectivity Analysis

To further validate the nature of reconstruction of the connectivity patterns for various ROIs, we performed a seed-based correlation analysis using the mean FC matrices predicted from the three models. We chose the left posterior cingulate cortex (PCC) as a seed region since it has been known to have an important functional role as a hub region of the default mode network^33^. Figure 3 plots the correlation values between left PCC and all other regions on the brain surface reconstructed from the Desikan-Killiany atlas^34^. Cool (hot) colors suggest low (high) connectivity (correlation) of that particular region with the left PCC (shown as dark red color in the mean empirical FC). The MKL model could reconstruct the connectivity pattern with higher precision than the other two models. It appears that due to very high correlation of left PCC with all other regions, DMF model could not as clearly distinguish the boundary between regions. SDK model could not possibly distinguish them due to very sparse connectivity between left PCC and all other regions.

**Figure 4:**
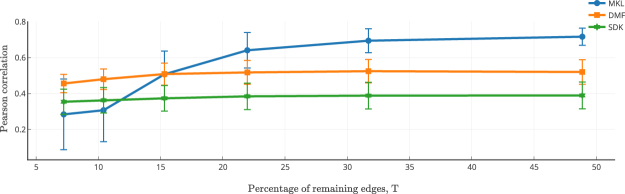
Model Performance on Sparse/Thresholded SCs. Structural Connectivity (SC) matrices for each subject were sparsified using 9 sparsification values. The three models were tested using these sparsified SCs. The plot shows the mean correlation values along with the standard deviation across subjects at each sparsification level. While MKL model was pre-trained with the un-sparsified SCs, a single optimal parameter was derived from the training data for each of the DMF and SDK models and used for estimating FC for the test subjects. The performance of MKL model starts to be superior after the sparsification level of 15% of the remaining edges. It appears that since MKL model selects the scales closely based on the SCs, the model performance degrades at very high sparsification levels.

### Effect of Thresholding

The rich-club organization of the structural connectivity (SC) matrix is previously demonstrated to be the backbone for generating the functional connectivity patterns^35–37^. Therefore we set out to investigate the impact of using thresholded SCs for predicting FCs. We pruned the SCs of all subjects by keeping only top T% of the connections (see Supplementary Section 1.4 for details). Each of these sparse matrices was passed as input to the learned MKL model. For each sparse SC, corresponding FC was predicted. Pre-learned *π*_*i*_′s were used for predicting FCs in the MKL model. Similarly, fixed diffusion scale and *G* parameters were used for SDK and DMF models for comparative evaluation. Figure 4 shows the mean correlation between empirical FC and predicted FCs for each of the sparse SC matrices. As can be seen, DMF and SDK models attain their respective optimum performance even when only few (as low as 10%) strongest edges in SC remained. On the other hand, MKL model requires both strong edges and few local edges, and hence its performance starts increasing from *T* = 15% and is significantly superior at all thresholds above this value. This result suggests that functional patterns may be primarily decided by the initial co-activations captured by *π*_*i*_′s and that the structural constraints of individual SCs provide paths for these activities to diffuse, giving support to our hypothesis. Nevertheless, stable performance with sparsification as high as with *T* = 20% indicates that all the models obey the basic rich-club principle.

**Figure 5:**
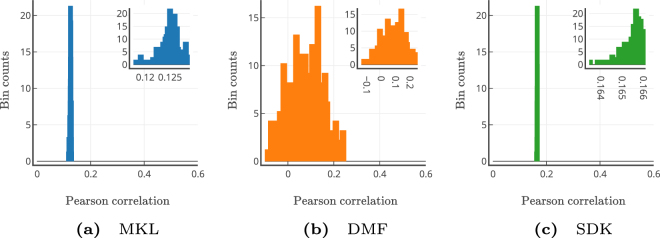
Model Performance with Perturbed Structural Connectivity (SC) Matrices. Randomly perturbed SCs (*N* = 250 sets) of the test subjects were used for estimating the FCs with the trained models. Sub-figures depict histograms of model’s mean performance (Pearson correlation between empirical and predicted FCs): (**a**) MKL; (**b**) DMF; and (**c**) SDK model, respectively. The sub-plots (top right corners) within these sub-figures zoom in on the histograms for clarity. As expected, all the models depict degradation in performance with perturbed SCs.

Interestingly, MKL model captures the differences in sparsity levels better when compared to the other two models, especially when SC was pruned to keep the strongest edges between 10–20%. This behavior suggest that pre-trained *π*_*i*_′s in MKL model do not compensate for major loss of information in sparsified SCs, thereby indicating avoidance of overfitting. Please see Supplementary Section 1.4 for the exact threshold values for this procedure.

### Robustness of the MKL Model

**Figure 6:**
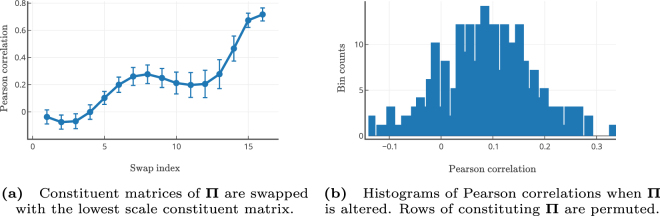
Investigation of the Impact of Altering the Scale-specificity of the Parameters *π*′s. Two studies are conducted where the first study (**a**) looks at the impact of changing the scale-specificity of the *π*′s and the second study (**b**) looks at the impact of a larger-scale alteration when the components of individual *π* matrices are themselves altered. (**a**) This sub-figure depicts the result of swapping each of the *π*_*i*_ matrices with the last matrix, i.e., with *π*_16_. For example, the first data point shows the mean performance when *π*_1_ is swapped with *π*_16_, the second data point corresponds to the case when *π*_2_ is swapped with *π*_16_ and so on for each of the 16 *π*_*i*_ matrices being swapped with the last matrix *π*_16_. Thus the last data point corresponds to the case when the original order was retained. The error bars represent the standard deviation. The results suggest scale-specificity of the learned parameters, i.e., in the sense that the performance degrades drastically if the *π* matrices of one scale are swapped with a *π* matrix of a distant scale. (**b**) The histogram of Pearson correlations depicts the performance when all the *π*_*i*_ matrices are stacked together and the rows of the resulting stacked Π-matrix are swapped randomly 250 times. Such global alteration drastically degrades the performance. Together, these results indicate that the learned parameters do not predict FCs by chance but play a crucial role in the MKL model.

The proposed model learns a latent representation, Π that maps the relationship between SC and FC. This being the crucial difference between MKL and other models, we performed extensive robustness tests to verify the usefulness of learning the *π*_*i*_′s. To ascertain that the model’s representation learns important features and does not capture the SC-FC mapping by chance, we conducted the following four randomization experiments. In the first one we randomize the input to the model (i.e., SCs) (see Fig. 5) and in the second the learning itself is conducted based on perturbed SCs (see Supplementary Fig. S5). In the third experiment we disturb the scale-specific relation between the learned *π*_*i*_′s and *H*_*i*_′s (see Eq. 21) and finally in the fourth experiment the constituent rows of ***π***_*i*_′s are randomly permuted (see Fig. 6).

#### Perturbing the model input

To verify whether the model learns the SC-FC relationship correctly or predicts the average FC independent of SC, we provided the MKL model with perturbed SCs in two possible scenarios: first, while testing, and second, while training.

Each subject-wise SC was perturbed *N* = 250 times, hence forming 250 sets of subject-wise perturbed SC-empirical FC pairs (please see Supplementary section 1.5 for perturbation procedure). In the first perturbation analysis, we trained the MKL model with the original subject-wise training SC-FC pairs, and tested the model with these 250 perturbation sets. These same sets were used for evaluating the other two models. We calculated the mean correlation values between predicted and empirical FCs, thus obtaining 250 mean correlation values for every model. Figure 5(a)–(c) show the histograms of these mean correlation values for MKL, DMF and SDK models, respectively. As expected, all the three models have significant drop in their performance indicating their sensitivity towards meaningful SC matrices while arriving at predictions.

In the second perturbation analysis, we trained *N* = 250 MKL models using the 250 purtubed sets and evaluated them using the subject-wise empirical SC-FC pairs. We did not have to perform this analysis for the other two models as this analysis is the same as that of the above for these models. Supplementary Fig. S5 shows the histogram of the 250 mean correlation values that is distributed across a wide range of correlation values instead of peaking at a particular value, thus indicating a *garbage-in, garbage-out* phenomenon from a machine learning perspective! This result, along with the results in Fig. 4, demonstrate that MKL model is not learning just a transformation from a subgraph of SC to an average FC but that the learning is holistic.

#### Altering the model parameters

After confirming that the model does not learn a random mapping between SC-FC pairs, we alter the learned mapping to further confirm model’s robustness. We considered two ways of altering the model parameters (*π*_*i*_′s). These parameters are mathematically represented as a set of *m* matrices (*π*_*i*_′s) corresponding to *m* diffusion scales (*m* here is set to 16, also see Eq. 21). We sought to experimentally verify that **Π** can be interpreted as holding complementary information of a cohort of SCs. Hence it is likely that any perturbation of **Π** would disturb the synergistic correspondence to empirical SCs and cause performance degradation. In order to experimentally validate this intuition, we ran two types of permutation tests.

Firstly, we sought to estimate the importance of the arrangement of ***π***_*i*_′s, i.e., the ordering of the scale-specific matrices constituting **Π**. For this we swapped every matrix *π*_*i*_ (1 ≤ *i* ≤ *m*) one at a time with ***π***_*m*_ (corresponding to the lowest scale, i.e., *π*_16_). Figure 6(a) shows the mean correlation while performing swapping. Pearson correlations are plotted against the swapped indices. Because of no-swap the last correlation (corresponding to *i* = 16) depicts optimal performance. This plot suggests that indeed matrices have positional significance (in other words, scale-specificity), so they cannot be reorganized to predict FC. This is a property that is also subtly captured in Eq. (21) in the sense that these matrices have a strict correspondence to their scales, consequently they embed scale-specific diffusion kernels to enable correct prediction of FC.

Secondly, we sought to estimate regional importance of the entries of *π*_*i*_ matrices across scales. We concatenate all *m π*_*i*_′s into a single matrix (**Π**) of size *mn* × *n*. We permute the rows of this matrix and test the model performance. A row of **Π** captures regional co-activations at that scale between the region corresponding to that row and all other regions. We permute the rows of this large matrix 250 (*N*). Each newly generated **Π** is used for testing the model performance. Figure 6(b) shows the histogram of the mean correlations of all the *N* permutations. Clearly the plot shows that permuted **Π** significantly deteriorates the model performance. This figure underlines the importance of maintaining the structure of co-activation between pairs of regions.

## Discussion

The holy grail in cognitive neuroscience is understanding how the static brain structure gives rise to dynamic function both during rest and task conditions. Several models have been proposed to characterize the structure-function relationship^38^. Simple linear diffusion models^18,19^ as well as complex non-linear, whole-brain computational models^32^ have been proposed. Linear graph models^18^ admit closed form deterministic and testable solution to macroscopic interactions of brain activity without requiring any details of neural coding or their biophysical substrate. On the other hand nonlinear complex drift-diffusion models based on excitatory and inhibitory neuronal populations, though not analytically tractable, give rise to rich dynamics^32^.

Abdelnour *et al*.^18^ conceived a model of functional connectivity (FC) with only one diffusion kernel defined at an optimal scale. This optimal kernel operates on an identity matrix, meaning that the amount of activity reaching other regions from a single source is representative of the statistical dependence between those regions. This statistical dependence resembles activity heat maps which exhibit inter-individual variations. However, Surampudi *et al*.^29^ showed that single kernel models do not generalize to a larger cohort and demonstrated that FC can be decomposed into multiple diffusion kernels with subject non-specific combination coefficients.

In this work, we proposed a *multiple kernel learning* (MKL) method that learns inter-regional co-activations (denoted as *π*_*i*_′s) and reshapes the structurally confined diffusion kernels to give rise to functional connectivity estimates. MKL model is a generalization of the SDK diffusion model (see Supplementary and Fig. S1 for the strengths and limitations of SDK models). Resting state functional connectivity could be considered as a signal on a brain graph expressed at multiple different spatio-temporal scales. Our approach essentially finds a way to unfold these solutions on the brain graph combining multiple scales to accurately estimate the empirical FC. One way to interpret the proposed multi-scale diffusion model is to treat it as a variant of a reaction-diffusion system on the graph determined by the underlying structural connectivity (SC) matrix.

In this work, we adopt the representation of the graph signal in terms of eigenvectors of the graph Laplacian similar to what has been recently proposed^28^. The proposed MKL framework devises a scheme for learning the hidden parameters (*π*_*i*_′s) to estimate FC. The initial regional activity *u*_0_ in the reaction-diffusion type model is a vector, hence the matrix ${u}_{0}{u}_{0}^{{\rm T}}$ is a rank 1 matrix. As it is a positive semi-definite (PSD) matrix, it will only have one non-zero eigenvalue. Eigen-decomposition in Eq. (11) suggests a possible physical interpretation, that the initial mean activity distribution, an eigenvector of the graph Laplacian, resembles standing wave patterns on the graph. Total number of such standing waves is equal to the number of nodes of the graph. Hence our hypothesis is that the initial regional co-activations (*π*_***i***_′s) correspond to one of the standing waves present at some time *k*_*i*_*τ* significantly changing the pattern at that reaction instance (please refer to section titled *Methods/Proposed MKL model* for notations). Functional connectivity can then be articulated as a superposition of such standing wave patterns and their regional co-activations.

In order to predict FC from the proposed diffusion model, we estimated **Π** by solving a LASSO optimization formulation. We hypothesized that these hidden parameters are learnable from the training data and remain fixed at the time of testing. Consequently different FC matrices for the test subjects would be arising by virtue of the underlying differences in the respective structural connectivity matrices (SCs). This would mean that the parameters **Π** are not merely a derivative of SCs but instead they complement the missing aspects by capturing the statistical dependence between two regions that are modulated by some intermediate region that may not be in physical proximity and that too operating at multiple resolutions or scales. Thus by incorporating the inter- and intra-hemispheric functional connectivity terms for a brain region, the learned optimal **Π** parameters enable more accurate matching of the structure-function correlation. All the computational models can be visualized to lie on the spectrum spanned by biological interpretability and analytical ease. Whereas linear models enjoy simplicity of solution of their models, non-linear models tend to explain the complex biological reality. MKL model seems to find a sweet spot and enjoys best of both by analytically providing the solution and explaining the patterns in terms of large-scale excitatory-inhibitory interactions. Since LASSO optimization is the most expensive computational step, the computational complexity of the proposed MKL model would be dominated by the cost of LASSO optimization.

In summary, on the model continuum, the proposed MKL model lies somewhere between simple linear diffusion models^18,19^ and complex non-linear drift diffusion models^32^. Consequently, we compared our simulation results predicting BOLD functional connectivity using the proposed model with models at either end of the complexity spectrum. The experimental results showed that the correlation structure of BOLD functional resting state brain networks is significantly well captured by our model. Prediction accuracy of the MKL model for the 23 test subjects is close to 0.70 whereas that of the non-linear model comes second best at 0.52 and that of the SDK model around 0.37. We conducted a series of tests that perturbed the inputs to the model as well as permuted the learned parameters **Π**. The test results attest to the robustness of the proposed model. Interestingly the model not only captures the variability of scales across participants but also demonstrates a possible application in characterizing age-related differences in learning optimal parameters for the accurate estimation of FC (refer to Supplementary section 4 and Figs S8–S10). Even in the face of considerable amount of variability present in the data, the proposed MKL model is still able to predict subject-specific FCs with high accuracy. Beyond this, functional connectivity subsumes the influence of different regions across scales and age groups providing a viability of **Π** being a useful parameter for classification purposes for other domains of application in health and disease. Overall, our method might be considered the missing link in the estimation and improvement of predicting subject-specific resting-state functional connectivity that remained elusive so far for complex non-linear and linear models. Given the strength of the analytical approach and tractability, the proposed model could be a suitable method for predicting task-based functional connectivity across different age groups.

One major limitation of our work is that it is not so straightforward like the linear diffusion model to invert the FC to recover the SC matrix. Currently, in the MKL model the procedure to predict SC from FC would rely on a given **Π**. One way of finding SC is by estimating the diffusion kernels for individual subjects by solving the same system of linear equations used to find FC. Laplacian of a graph could then be estimated. Carefully recovering multiple diffusion kernels might turn out to cause numerical instability to the Laplacian (see Supplementary section 5 for details of the proposed inversion) and this issue needs to be resolved in the future. While in the current formulation we are empirically determining the number of scales (*m*) and their spacing, optimization formulation could be modified to estimate these automatically. Moreover, the current model does not consider the non-stationary nature of functional connectivity, the so called functional connectivity dynamics (FCD). Future studies can look at optimization procedures of MKL for modeling the dynamic functional connectivity which is more realistic than modeling stationary FC.

## Methods

### Notations

This section introduces the notations used here as well as in the Supplementary material. Please refer to Table 1 for all the notations.

**Table 1:** Notations used in the models and optimization formulation.

Object	Description
n	Number of ROIs or the number of nodes in the brain graph.
p	Number of training subjects.
SC	Structural connectivity matrix.
SC^*s*^	SC matrix for subject *s*.
D^*s*^	Degree matrix for subject *s*; sum of edge weights for every region.
FC	Functional connectivity matrix.
FC^*s*^	FC matrix for subject *s*.
	$$[{f}_{1}^{s},\cdots ,{f}_{n}^{s}]$$
**W**_*n*×*n*_	Weighted adjacency matrix of a graph.
**D**_*n*×*n*_	Degree matrix of a graph, computed by taking the sum of all weights on every node and diagonalizing the vector.
${{\bf{L}}}_{n\times n}^{s}$	Laplacian matrix of subject *s*.
${{\rm{\Psi }}}_{n\times n}^{s}$	Eigenvector matrix of the graph Laplacian of subject *s*.
${{\rm{\Lambda }}}_{n\times n}^{s}$	Eigenvalue matrix, diagonal matrix with increasing order of eigenvalues, of the graph Laplacian of subject *s*.
*γ*_*i*_	A scale at which diffusion kernel is defined.
${{\bf{H}}}_{i\,n\times n}^{s}$	Diffusion kernel at scale *γ*_*i*_ for subject *s*.
*m*	Number of scales
${{\bf{H}}}_{n\times mn}^{s}$	Collection of all *m* diffusion kernels of a subject *s*. $$[\begin{array}{ccc}{{\bf{H}}}_{1\,n\times n}^{s} & \cdots & {{\bf{H}}}_{m\,n\times n}^{s}\end{array}]$$
π_*in*×*n*_	Interregional co-activations corresponding to scale *γ*_*i*_.
**Π**_*mn*×*n*_	Interregional co-activations collectively represented at all scales. $$[\begin{array}{c}{\pi }_{1n\times n}\\ \vdots \\ {\pi }_{mn\times n}\end{array}]=[\begin{array}{ccc}{{{\rm{\Pi }}}^{1}}_{mn\times 1} & \cdots & {{{\rm{\Pi }}}^{n}}_{mn\times 1}\end{array}]$$
***X***_*pn*×*mn*_	$$[\begin{array}{c}{{\bf{H}}}^{1}\\ \vdots \\ {{\bf{H}}}^{p}\end{array}]$$
***Y***_*pn*×*n*_	$$[\begin{array}{c}{{{\rm{FC}}}^{1}}_{n\times n}\\ \vdots \\ {{{\rm{FC}}}^{p}}_{n\times n}\end{array}]=[\begin{array}{ccc}{f}_{1}^{1} & \cdots & {f}_{n}^{1}\\ & \vdots & \\ {f}_{1}^{p} & \cdots & {f}_{n}^{p}\end{array}]=[\begin{array}{ccc}{Y}_{1pn\times 1} & \cdots & {Y}_{npn\times 1}\end{array}]$$
${{\bf{C}}}_{f}^{s}$	Predicted FC ${\sum }_{i=1}^{m}{{\bf{H}}}_{i}^{s}{\pi }_{i}$
${{\bf{C}}}_{f}{|}_{{k}_{0}}$	Functional connectivity FC when reaction only happens at *k*_0_*τ*.

### Proposed Multiple Kernel Learning (MKL) Model

In this section we describe the proposed model as a variant of Reaction-diffusion (RD) systems^39^ wherein the regional mean activities diffuse on the graph determined by anatomical pathways (SC). RD systems explain the formation of complex self-organizing patterns naturally occurring in nature^40^. RD systems have been employed to model interaction among populations of neurons and the emerging patterns of functional connectivity among neural ensembles^41–44^. Just as statistical thermodynamics relates brownian motion of fluid particles to mean motion of a whole fluid, Wilson-Cowan equations characterize the macro-scopic statistical behavior of mean fields of the resulting neural activities^26,45^. Atasoy *et al*.^28^ embed anatomical constraints in terms of the graph Laplacian matrix of the SC matrix in the Wilson-Cowan equations to explain the macro-scale excitatory and/or inhibitory interactions of the regional activities. These excitatory and/or inhibitory interactions result in the formation of complex functional patterns such as RSNs. We extend our model from Atasoy *et al*.^28^ and explain the formation of FC through RSNs. We hypothesize that the cumulative mean activities of all the regions is generated by intra-regional micro-scale dynamics which diffuses inter-regionally on the structural connectome. We propose a physical model that implicitly captures the pairwise functional interactions between ROIs by explicitly associating them with their extent of influence through the diffusion kernels on the SC (see Supplementary section 3 for details on graph Laplacian and diffusion kernels).

The derivation of the expression for FC consists of five major stages. We consider that FC matrix encompasses effects of diffusion from multiple reactions. In the first stage, we formulate the differential equation for the time evolution of regional activities (Eqs 1–3). In second stage, we characterize the time evolution of the regional activities in an arbitrarily small time interval (Eqs 5 and 6). In the third stage, we integrate the diffusion process over all the connectome harmonics (Eqs 7 and 8). In the fourth stage we accumulate the diffusions happening in various time intervals to generate the complete expression for FC (Eqs 9–21). This FC assumes the form of a combination of diffusion kernels weighted by scale-specific parameters (Eq. 21). In the final stage, we propose an optimization framework for estimating these global parameters (Eqs 22–27).

Let the cumulative mean activities for all regions be denoted by **u**(*t*)_*n*×1_ at time *t*. We assume that these activities belong to either excitatory and/or inhibitory interactions. The temporal evolution of regional activities are modeled as the following linear variant of Wilson-Cowan equations:

1 $$\tau \frac{\partial }{\partial t}{\bf{u}}(t)=-{\bf{u}}(t)+{\mathscr{D}}[{\bf{u}}(t\mathrm{)]}.$$

where ${\mathscr{D}}$ is the spatial propagation operator. *τ* is a characteristic time scale that speeds up or slows down the evolution of the system. Mean activity of a region *i*, *u*_*i*_(*t*), can be abstracted out from biological details as a one-dimensional (1-D) time varying signal. A vector of these 1-D signals indexed by the nodes of the graph represents a *graph signal*. We represent the graph signal in terms of its Fourier components using graph Fourier transform^46^:

2 $${\bf{u}}(t)={\rm{\Psi }}\beta (t),$$

where Ψ is the eigenvector matrix of graph Laplacian (see Supplementary section 3) and *β* (*t*) is its Fourier representation at time *t*. With this decomposition temporal dynamics is explicitly represented using spatial basis functions. Further we conceptualize the spatial operator ${\mathscr{D}}$ in the form of a diffusion kernel defined at scale *σ*^2^/2 on the structural brain graph Laplacian (Λ) corresponding to the time interval *τ* between two consecutive reaction instances.

3 $${\mathscr{D}}[{\bf{u}}(t)]={\rm{\Psi }}{e}^{-{\rm{\Lambda }}{\sigma }^{2}/2}{{\rm{\Psi }}}^{{\rm{{\rm T}}}}{\bf{u}}(t)={\rm{\Psi }}{e}^{-{\rm{\Lambda }}{\sigma }^{2}/2}\beta (t).$$

Substituting Equations (2) and (3) and combining the fact that Ψ is invertible, differential Equation (1) can be solved for *β* (*t*) which represents the signal evolution in the time interval between two reaction instances, as follows:

4 $$\tau {\rm{\Psi }}\frac{d}{dt}\beta (t)=-{\rm{\Psi }}\beta (t)+{\rm{\Psi }}{e}^{-{\rm{\Lambda }}{\sigma }^{2}/2}\beta (t)$$

5 $$\frac{d}{dt}\beta (t)=-\frac{1}{\tau }({{\bf{I}}}_{n}-{e}^{-{\rm{\Lambda }}{\sigma }^{2}/2})\beta (t)$$

6 $$\beta (t)={e}^{-1\tau ({{\bf{I}}}_{n}-{e}^{-{\rm{\Lambda }}{\sigma }^{2}/2})t}{\beta }_{0},$$

where *β*_0_ represents the initial mean activity. Equation (6) depicts how the mean activity (*β*_0_) of every region diffuses on the graph. Finally the graph signal between two reaction times can be expressed in a closed form (substituting Equation (6) in (2)) as:

7 $$\begin{array}{rcl}{\bf{u}}(t) & = & {\rm{\Psi }}{e}^{-1/\tau ({{\bf{I}}}_{n}-{e}^{-{\rm{\Lambda }}{\sigma }^{2}/2})t}{\beta }_{0}\end{array}$$

8 $$\begin{array}{rcl} & = & {\rm{\Psi }}{e}^{-1/\tau ({{\bf{I}}}_{n}-{e}^{-{\rm{\Lambda }}{\sigma }^{2}/2})t}{{\rm{\Psi }}}^{{\rm T}}{{\bf{u}}}_{0}.\end{array}$$

where, **u**_0_ = Ψ*β*_0_ captures initial activity just after reaction, or at the start of diffusion. **u**_0_ depends on the magnitude of reaction phenomenon, hence may change after every reaction instance. Given the temporal evolution of graph signal, we will next derive how this leads to the evolution of functional connectivity and RSNs. RSNs have unique correspondence with graph-harmonics/eigenvectors of the structural graph Laplacian^28^. We develop the model for a single graph-harmonic, i.e., for all RSNs corresponding to that graph-harmonic. Finally, we superpose all the patterns of the resting state networks and explain the formation of FC.

The graph signal **u**_0_ may not change significantly in every reaction. Equation (8) represents the diffusive phenomenon of the graph signal over the characteristic time *τ*. Let **u**_0_ change significantly at scalar multiples of *τ*, i.e., *t* + *k*_0_*τ*, *t* + *k*_0_*τ* + *k*_1_*τ*, … with corresponding amplitudes *a*_0_, *a*_1_, …, respectively. For now we consider generating the functional connectivity $({{\bf{C}}}_{f}{|}_{{k}_{0}})$ for the time interval between two consecutive reactions; at times *t* + *k*_0_*τ* and *t* + *k*_0_*τ* + *k*_1_*τ*.

9 $$\begin{array}{rcl}{{\bf{C}}}_{f}{|}_{{k}_{0}} & = & {\int }_{t+{k}_{0}\tau }^{t+{k}_{0}\tau +{k}_{1}\tau }{\bf{u}}(t){\bf{u}}{(t)}^{{\rm{{\rm T}}}}dt\end{array}$$

10 $$\begin{array}{rcl} & = & {\int }_{t+{k}_{0}\tau }^{t+{k}_{0}\tau +{k}_{1}\tau }{\rm{\Psi }}{e}^{-1/\tau ({{\bf{I}}}_{n}-{e}^{-{\rm{\Lambda }}{\sigma }^{2}/2})t}{{\rm{\Psi }}}^{{\rm{{\rm T}}}}({a}_{0}{{\bf{u}}}_{0})({a}_{0}{{\bf{u}}}_{0}^{{\rm{{\rm T}}}}){\rm{\Psi }}{e}^{-1/\tau ({{\bf{I}}}_{n}-{e}^{-{\rm{\Lambda }}{\sigma }^{2}/2})t}{{\rm{\Psi }}}^{{\rm{{\rm T}}}}dt.\end{array}$$

As **u**_0_ is also a signal on graph, we can express the positive semi-definite (PSD) matrix ${{\bf{u}}}_{0}{{\bf{u}}}_{0}^{{\rm{{\rm T}}}}$ in terms of its eigen-decomposition. And as it is only a single harmonic, Δ is a diagonal matrix with only one non-zero entry.

11 $${{\bf{u}}}_{0}{{\bf{u}}}_{0}^{{\rm{{\rm T}}}}={{\rm{\Psi }}{\rm{\Delta }}{\rm{\Psi }}}^{{\rm{{\rm T}}}}.$$

Hence, ${{\bf{C}}}_{f}{|}_{{k}_{0}}$ takes the following form:

12 $$\begin{array}{rcl}{{\bf{C}}}_{f}{|}_{{k}_{0}} & = & {a}_{0}^{2}{\int }_{t+{k}_{0}\tau }^{t+{k}_{0}\tau +{k}_{1}\tau }{\rm{\Psi }}{e}^{-1/\tau ({{\bf{I}}}_{n}-{e}^{-{\rm{\Lambda }}{\sigma }^{2}/2})t}{\rm{\Delta }}{e}^{-1/\tau ({{\bf{I}}}_{n}-{e}^{-{\rm{\Lambda }}{\sigma }^{2}/2})t}{{\rm{\Psi }}}^{{\rm{{\rm T}}}}dt\end{array}$$

13 $$\begin{array}{rcl} & = & {a}_{0}^{2}{\int }_{t+{k}_{0}\tau }^{t+{k}_{0}\tau +{k}_{1}\tau }{\rm{\Psi }}{e}^{-2/\tau ({{\bf{I}}}_{n}-{e}^{-{\rm{\Lambda }}{\sigma }^{2}/2})t}{{\rm{\Delta }}{\rm{\Psi }}}^{{\rm{{\rm T}}}}dt\end{array}$$

14 $$\begin{array}{rcl} & = & {a}_{0}^{2}{\int }_{t+{k}_{0}\tau }^{t+{k}_{0}\tau +{k}_{1}\tau }\{{\rm{\Psi }}{e}^{-2/\tau ({{\bf{I}}}_{n}-{e}^{-{\rm{\Lambda }}{\sigma }^{2}/2})t}{{\rm{\Psi }}}^{{\rm{{\rm T}}}}\}\{{{\rm{\Psi }}{\rm{\Delta }}{\rm{\Psi }}}^{{\rm{{\rm T}}}}\}dt\end{array}$$

15 $$\begin{array}{rcl} & = & {\rm{\Psi }}{\int }_{t+{k}_{0}\tau }^{t+{k}_{0}\tau +{k}_{1}\tau }{e}^{-2/\tau ({{\bf{I}}}_{n}-{e}^{-{\rm{\Lambda }}{\sigma }^{2}/2})t}dt{{\rm{\Psi }}}^{{\rm{{\rm T}}}}\{{a}_{0}^{2}{\theta }_{{k}_{0}}\}.\end{array}$$

We can denote the initial activity matrix as ${a}_{0}^{2}{\theta }_{{k}_{0}}$. As reaction instances are not usually far apart in time, instead of double exponentiation we utilize the first order Taylor approximation for the exponent of the integrand; i.e. ${{\bf{I}}}_{n}-{e}^{-{\rm{\Lambda }}{\sigma }^{2}/2}\approx {\rm{\Lambda }}{\sigma }^{2}/2$. Hence, ${{\bf{C}}}_{f}{|}_{{k}_{0}}$ becomes

16 $${{\bf{C}}}_{f}{|}_{{k}_{0}}=({\rm{\Psi }}{e}^{-{\sigma }^{2}/\tau {\rm{\Lambda }}(t+{k}_{0}\tau )}{{\rm{\Psi }}}^{{\rm{{\rm T}}}}-{\rm{\Psi }}{e}^{-{\sigma }^{2}/\tau {\rm{\Lambda }}(t+{k}_{0}\tau +{k}_{1}\tau )}{{\rm{\Psi }}}^{{\rm{{\rm T}}}})({a}_{0}^{2}\tau /{\sigma }^{2}{{\rm{\Lambda }}}^{-1}{\theta }_{{k}_{0}}).$$

We call the matrix independent of time as

17 $${\pi }_{0}={a}_{0}^{2}\tau /{\sigma }^{2}{{\rm{\Lambda }}}^{-1}{\theta }_{{k}_{0}}$$

Now with multiple reactions happening at multiples of *τ*, we sum over all the reaction instances to get the functional connectivity matrix:

18 $${{\bf{C}}}_{f}=\sum _{i}{\rm{\Psi }}{e}^{-{\sigma }^{2}/\tau {\rm{\Lambda }}{k}_{i}}{{\rm{\Psi }}}^{{\rm{{\rm T}}}}{\pi }_{i},$$

where,

19 $${\pi }_{i}=({a}_{i}^{2}-{a}_{i-1}^{2})\tau /{\sigma }^{2}{{\rm{\Lambda }}}^{-1}{\theta }_{{k}_{i}}.$$

Observing the structure of the FC matrix, FC is conceptualized as being represented by diffusion kernels and their corresponding inter-regional mean activities. So, the larger the value of *k*_*i*_, the lesser is its contribution to FC. This means that summation on a finite number of diffusion scales is sufficient for reproducing FC (in this work we considered 16 diffusion scales based on pilot simulations). Now after combining the functional patterns of all the graph-harmonics, we approximate empirical FC with *m* number of diffusion scales *γ*_*i*_′s. The model thus takes the form as follows:

20 $$\begin{array}{rcl}{{\bf{C}}}_{f} & = & \sum _{i=1}^{m}{\rm{\Psi }}{e}^{-{\rm{\Lambda }}{\gamma }_{i}}{{\rm{\Psi }}}^{{\rm{{\rm T}}}}{\pi }_{i}\end{array}$$

21 $$\begin{array}{rcl} & = & \sum _{i=1}^{m}{{\bf{H}}}_{i}{\pi }_{i}.\end{array}$$

where, **H**_*i*_ denotes the diffusion kernel at scale *γ*_*i*_. Further the model in Equation (21) suggests that the scale of diffusion is determined by a characteristic time constant (*τ*), spatial diffusion variance (*σ*^2^) and the time interval between consecutive reaction instances. Matrix *π*_*i*_ represents the scale-specific initial relationships in the mean regional activities.

Proposed model represents the functional connectivity in terms of diffusion kernels operating on scale-specific matrices. It can be inferred that Adelnour *et al*.^18^ envisage FC comprising only one diffusion kernel defined at an optimal scale. The optimal kernel operates on an identity matrix; meaning only the concerned region has non-zero mean activity independent of other regions, i.e., the amount of activity reaching other regions from the single source is representative of the statistical dependence between those two regions. Surampudi *et al*.^29^ demonstrated that FC can be decomposed into multiple diffusion kernels whose combination coefficients are unique to the cohort. In addition to the multiple scales, proposed model provides inter-regional relationships instead of individually active regions. The proposed model generalizes both the aforementioned models as statistical dependence between two regions may be modulated by some intermediate regions without physical proximity that too at multiple resolutions or scales. Moreover, the model provides a biological interpretation of the diffusion scales and has an organic relationship to the reaction-diffusion system.

### Optimization formulation

We hypothesize that the global parameters *π*_*i*_′s are estimated from the training subjects (indexed by *s* and varies from 1 to *p*) and remain fixed for all the test subjects. In order to estimate *π*_*i*_′s we utilize an optimization formulation that minimizes an objective function *J* comprising the mean squared error between empirical and predicted FCs.

22 $$\begin{array}{rcl}{\bf{J}} & = & \sum _{s=1}^{p}{\Vert {{\bf{C}}}_{f}^{s}-{{\rm{FC}}}^{s}\Vert }_{F}^{2}\end{array}$$

23 $$\begin{array}{c}\begin{array}{rcl} & = & \sum _{s=1}^{p}{\Vert \sum _{i=1}^{m}{{\bf{H}}}_{i}^{s}{\pi }_{i}-{{\rm{FC}}}^{s}\Vert }_{F}^{2}\end{array}\end{array}$$

24 $$\begin{array}{rcl} & = & {\Vert \sum _{s\mathrm{=1}}^{p}\sum _{j\mathrm{=1}}^{n}{{\bf{H}}}^{s}{{\rm{\Pi }}}^{j}-{f}_{j}^{s}\Vert }_{F}^{2}\end{array}$$

25 $$\begin{array}{rcl} & \le  & \sum _{j=1}^{n}{\Vert \sum _{s=1}^{p}{{\bf{H}}}^{s}{{\rm{\Pi }}}^{j}-{f}_{j}^{s}\Vert }_{F}^{2}.\end{array}$$

To keep the number of reacting regions less, we employ *L*_1_ norm on **Π**^*j*^′s.

26 $${\bf{J}}\le \sum _{j=1}^{n}{\Vert \sum _{s=1}^{p}{{\bf{H}}}^{s}{{\rm{\Pi }}}^{j}-{f}_{j}^{s}\Vert }_{F}^{2}+\lambda {\Vert {{\rm{\Pi }}}^{j}\Vert }_{1}$$

27 $$=\sum _{j=1}^{n}{\Vert X{{\rm{\Pi }}}^{j}-{Y}_{j}\Vert }_{F}^{2}+\lambda {\Vert {{\rm{\Pi }}}^{j}\Vert }_{1}$$

We apply sparsity on each column and compute every column of **Π**^*j*^ separately. The objective function takes the form well known in regression analysis as *least absolute shrinkage and selection operator* (LASSO) that performs both variable selection and regularization. We passed the respective matrices into *lasso solver* to get the solution. We arrived at the model parameters experimentally, for example, the number of scales *m* is empirically chosen.

### DMF Model

We used the reduced dynamic mean field model as the non-linear model for comparative analysis^32^. This approach considers models with synaptic gating variable with passive decay differential equation along with Gaussian fluctuations. Firing rate was approximated based on input-output sigmoid function of the synaptic gating variable. The whole dynamics of each local network of excitatory and inhibitory populations of spiking neurons interconnected via excitatory synapses can be expressed by a single one-dimensional equation. The global brain dynamics of the network of interconnected local networks can be described by the following set of coupled non-linear stochastic differential equations^32^:

28 $$\frac{d{S}_{i}}{dt}=-\frac{{S}_{i}}{{\tau }_{S}}+(1-{S}_{i})\gamma H({x}_{i})+\sigma {\nu }_{i}(t)$$

29 $$H({x}_{i})=\frac{a{x}_{i}-b}{1-{\exp }(-d(a{x}_{i}-b))}$$

30 $${x}_{i}=w{J}_{N}{S}_{i}+G{J}_{N}\sum _{j}{C}_{ij}{S}_{j}+{I}_{0}$$

Here *S*_*i*_ is synaptic gating variable of area *i*. *x*_*i*_ is population mean firing rate for region *i*. *J*_*N*_ is the excitatory synaptic coupling. *ν*_*i*_ in (28) is uncorrelated standard Gaussian noise with noise amplitude *σ* = 0.001 nA. *I*_0_ is the external input current. *C*_*ij*_ represents entries of the SC matrix which captures the structural connectivity between regions *i* and *j*. Parameter values were selected as in Deco *et al*.^32^. A forward BOLD model was used that converts the local synaptic activity of a given cortical area into an observable BOLD signal. The simulated BOLD signal was down-sampled at 2 secs to have the same temporal resolution as in the empirically measured BOLD signal. Simulation length for computing the model FC was equivalent to 8 minutes. The coupling parameter *G* (see Equation (30)) is varied between 0 to 3. We use individual empirical SC - FC matrices for exploration of subject-wise parameters for optimal fit. The optimal *G* value varied among the subjects from 0.5 to 3. The mode of the distribution of the parameters obtained for training subjects was taken as the optimal *G*s for the training cohort and was found to be 2.85. The same value was used to estimate predicted FCs for all the test subjects.

### SDK model

We used a linear diffusion model described in Abdelnour *et al*.^18^. This model considers SC matrix of a participant as the weighted adjacency matrix and computes graph Laplacian (described in Supplementary section 3). Then it assumes a set of scales and defines diffusion kernels at each scale. Iteratively for each diffusion kernel, the estimated FC is compared with empirical FC of that subject in terms of Pearson correlation. The scale at which Pearson correlation was found to be maximum is considered subject-specific optimum scale and its corresponding diffusion kernel is hypothesized as FC. Amongst the training subjects, we found that the mode of the optimal diffusion scale was 0.8, and this was maintained as a fixed parameter for all the test subjects.

### Data availability statement

The datasets generated during and/or analysed during the current study are available from the corresponding author on reasonable request. The codes for the MKL model are available at https://github.com/govindasurampudi/MKL.

## Electronic supplementary material

Supplementary File

## References

[1] Hagmann, P. *From diffusion MRI to brain connectomics*. Ph.D. thesis, Université de Lausanne (2005).

[2] Sporns O, Tononi G, Kötter R (2005). The human connectome: a structural description of the human brain. PLoS Comput Biol.

[3] David O, Cosmelli D, Friston KJ (2004). Evaluation of different measures of functional connectivity using a neural mass model. Neuroimage.

[4] Ioannides AA (2007). Dynamic functional connectivity. Current opinion in neurobiology.

[5] Hutchison RM (2013). Dynamic functional connectivity: promise, issues, and interpretations. Neuroimage.

[6] Hutchison RM, Womelsdorf T, Gati JS, Everling S, Menon RS (2013). Resting-state networks show dynamic functional connectivity in awake humans and anesthetized macaques. Human brain mapping.

[7] Ryali S (2016). Temporal dynamics and developmental maturation of salience, default and central-executive network interactions revealed by variational bayes hidden markov modeling. PLOS Computational Biology.

[8] Cabral J, Kringelbach ML, Deco G (2012). Functional graph alterations in schizophrenia: a result from a global anatomic decoupling?. Pharmacopsychiatry.

[9] Bettinardi RG (2017). How structure sculpts function: Unveiling the contribution of anatomical connectivity to the brain’s spontaneous correlation structure. Chaos: An Interdisciplinary Journal of Nonlinear Science.

[10] Honey C (2009). Predicting human resting-state functional connectivity from structural connectivity. Proceedings of the National Academy of Sciences.

[11] Bressler SL, Menon V (2010). Large-scale brain networks in cognition: emerging methods and principles. Trends in cognitive sciences.

[12] Deco G, Kringelbach ML (2014). Great expectations: using whole-brain computational connectomics for understanding neuropsychiatric disorders. Neuron.

[13] Deco G, Rolls ET, Horwitz B (2004). “what” and “where” in visual working memory: a computational neurodynamical perspective for integrating fmri and single-neuron data. Journal of Cognitive Neuroscience.

[14] Newman SD, Carpenter PA, Varma S, Just MA (2003). Frontal and parietal participation in problem solving in the tower of london: fmri and computational modeling of planning and high-level perception. Neuropsychologia.

[15] Galán RF (2008). On how network architecture determines the dominant patterns of spontaneous neural activity. PloS one.

[16] Barnett L, Buckley CL, Bullock S (2009). Neural complexity and structural connectivity. Physical Review E.

[17] Hlinka J, Coombes S (2012). Using computational models to relate structural and functional brain connectivity. European Journal of Neuroscience.

[18] Abdelnour F, Voss HU, Raj A (2014). Network diffusion accurately models the relationship between structural and functional brain connectivity networks. Neuroimage.

[19] Saggio ML, Ritter P, Jirsa VK (2016). Analytical operations relate structural and functional connectivity in the brain. PloS one.

[20] Deco G, Jirsa VK, McIntosh AR (2011). Emerging concepts for the dynamical organization of resting-state activity in the brain. Nature reviews. Neuroscience.

[21] Deco G, Jirsa V, McIntosh AR, Sporns O, Kötter R (2009). Key role of coupling, delay, and noise in resting brain fluctuations. Proceedings of the National Academy of Sciences.

[22] Hahn G (2017). Spontaneous cortical activity is transiently poised close to criticality. PLOS Computational Biology.

[23] Nakagawa, T. T., Adhikari, M. H. & Deco, G. Large-scale computational models of ongoing brain activity. *Computational Models of Brain and Behavior* 425–437.

[24] Kuramoto, Y. *Chemical oscillations, waves, and turbulence*, vol. 19 (Springer Science & Business Media 2012).

[25] Wilson HR, Cowan JD (1972). Excitatory and inhibitory interactions in localized populations of model neurons. Biophysical journal.

[26] Wilson HR, Cowan JD (1973). A mathematical theory of the functional dynamics of cortical and thalamic nervous tissue. Biological Cybernetics.

[27] Destexhe A, Sejnowski TJ (2009). The wilson–cowan model, 36 years later. Biological cybernetics.

[28] Atasoy, S., Donnelly, I. & Pearson, J. Human brain networks function in connectome-specific harmonic waves. *Nature communications* **7** (2016). 10.1038/ncomms10340 PMC4735826 26792267

[29] Combining multiscale diffusion kernels for learning the structural and functional brain connectivity. *bioRxiv* 078766 (2016).

[30] Lanckriet GR, Cristianini N, Bartlett P, Ghaoui LE, Jordan MI (2004). Learning the kernel matrix with semidefinite programming. Journal of Machine Learning Research.

[31] Gonen M, Alpaydin E (2011). Multiple kernel learning algorithms. Journal of Machine Learning Research.

[32] Deco G (2013). Resting-state functional connectivity emerges from structurally and dynamically shaped slow linear fluctuations. Journal of Neuroscience.

[33] Damoiseaux J (2006). Consistent resting-state networks across healthy subjects. Proceedings of the national academy of sciences.

[34] Desikan RS (2006). An automated labeling system for subdividing the human cerebral cortex on mri scans into gyral based regions of interest. Neuroimage.

[35] Ven Den Heuvel MP, Sporns O (2011). Rich-club organization of the human connectome. Journal of Neuroscience.

[36] Collin G, Sporns O, Mandl RC, van den Heuvel MP (2014). Structural and functional aspects relating to cost and benefit of rich club organization in the human cerebral cortex. Cerebral cortex.

[37] Senden M, Deco G, de Reus MA, Goebel R, van den Heuvel MP (2014). Rich club organization supports a diverse set of functional network configurations. Neuroimage.

[38] Pillai AS, Jirsa VK (2017). Symmetry breaking in space-time hierarchies shapes brain dynamics and behavior. Neuron.

[39] Morrison, P. The undecidable: Basic papers on undecidable propositions, unsolvable problems and computable functions (1965).

[40] Camazine, S. *Self-organization in biological systems* (Princeton University Press, 2003).

[41] Isaacson JS, Scanziani M (2011). How inhibition shapes cortical activity. Neuron.

[42] Turing AM (1990). The chemical basis of morphogenesis. Bulletin of mathematical biology.

[43] Kondo S, Miura T (2010). Reaction-diffusion model as a framework for understanding biological pattern formation. Science.

[44] Angstmann CN, Donnelly IC, Henry BI (2013). Pattern formation on networks with reactions: A continuous-time random-walk approach. Physical Review E.

[45] Kilpatrick, Z. P. Wilson-cowan model. *Encyclopedia of Computational Neuroscience* 3159–3163 (2015).

[46] Shuman DI, Narang SK, Frossard P, Ortega A, Vandergheynst P (2013). The emerging field of signal processing on graphs: Extending high-dimensional data analysis to networks and other irregular domains. IEEE Signal Processing Magazine.

[47] Xia M, Wang J, He Y (2013). Brainnet viewer: a network visualization tool for human brain connectomics. PloS one.

[48] Rubinov M, Sporns O (2010). Complex network measures of brain connectivity: uses and interpretations. Neuroimage.

