# Supplementary material for: Multiple Kernel Learning Model for Relating Structural and Functional Connectivity in the Brain
Source: Sci Rep. 2018 Feb 19;8:3265. doi: 10.1038/s41598-018-21456-0 (PMC5818607; doi:10.1038/s41598-018-21456-0)
Supplement: Supplementary file 1 — Supplementary File [file 41598_2018_21456_MOESM1_ESM.pdf]

Supplementary Material
Multiple Kernel Learning Model for Relating Structural and Functional Connectivity in the Brain

Sriniwas Govinda Surampudi, Shruti Naik, Raju Bapi Surampudi,
Viktor K. Jirsa, Avinash Sharma, Dipanjan Roy

1 Parameter Determination and Analysis of the MKL Model

This section describes the practical aspects of training the model. We partitioned the subject pool into two sets - training and testing sets. LASSO method can optimize for one vector instead of a matrix, hence we trained individual columns of $\mathbf{\Pi}$ separately. Ascending order of scales focus on further local connections. i.e. scale index 1 corresponds to global diffusion phenomenon and scale index 16 corresponds to local diffusion phenomenon. Rest of the scale indices correspond to intermediate diffusion phenomena in sequence.

1.1 Choice of scales

A scale of a diffusion kernel represents the extent of spread of the graph signal from the source node where it is deployed. Depending on the graph topology, a fixed scale for all graphs will result in different extents of spread of the signal on individual graphs. Diffusion kernels capture the extent of spread with their scale parameter γ. Hence to normalize the spread across subjects in the cohort, we fixed the multiple spreads and selected graph (or subject) specific scales. Normalization of diffusion scales is very important as the diffusion scale value is relative to the graph structure/topology, a fixed value may not be suitable for all graphs. Therefore, we fix a set of exponential values α_i's which remain the same for all participants. Figure S2 shows the procedure for selecting the subject-specific scales, here for a subject. One fixed exponential value $f(\lambda, \gamma_i) = \alpha_i$ provides one diffusion scale. A diffusion scale is then calculated as:

$$\alpha_i = e^{-\lambda \gamma_i} \tag{1}$$

$$\gamma_i = {}^{-\ln(\alpha_i)}/_{\lambda}. \tag{2}$$

Exponential curves represent the 16 selected scales for a sample subject.

1.2 Choosing Sufficient Number of Scales

This subsection looks at the question of how many scales m would be sufficient for the proposed MKL model. This analysis also demonstrates the robustness of the MKL method with respect to the choice of the number of scales, i.e., the choice of the parameter m. Here we analyze with 5 different values of m using the same training and testing sets used to train the model. We ran the MKL model for each m separately and plotted box-plots for the same in figure S3. The yellow line passing through the boxes joins the mean performance of the models, dotted line. The figure suggests that model performance decreases as m changes from 2 to 4 and then increases till $m = 16$. Further decrease in performance suggests that model might start over-fitting on the training data for scales beyond 16.

1.3 Configuration of scales

Next we intend to see how important the arrangement of scales is, i.e., how the configuration of scales affects the prediction of FC. To show that the chosen scale set is close to optimal, we randomly generated several

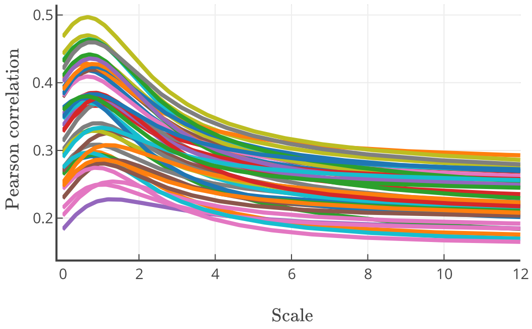

(a) Pearson correlation curves with thresholding

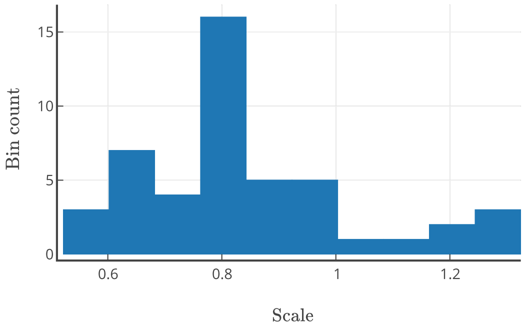

(b) Distribution of scales with thresholding

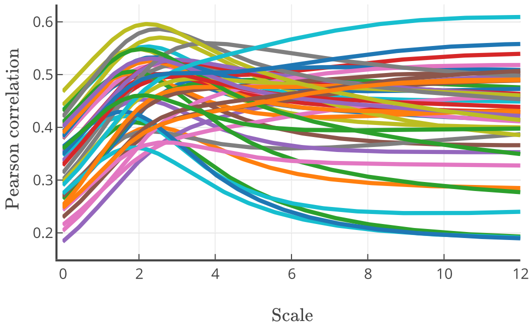

(c) Pearson correlation curves without thresholding

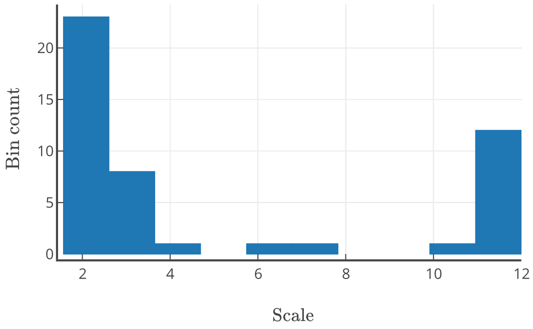

(d) Distribution of scales without thresholding

Figure S1: **Variation in the Optimum Scale across all Subjects in the Single Scale Diffusion Kernel (SDK) Model.** We simulated the SDK model for the Berlin dataset and found that there is a variation in the subject-wise optimum scale. When the SC matrices are pruned by removing edges less than 0.07% of the maximum edge value (in the adaptation of Abdelnour et al.'s implementation code [[1]]), there seems to be a unique scale for the cohort. (a) shows the Pearson correlation curves for all the subjects. The histogram of the optimum subject-specific scales also has some variations as shown in (b). But, when the SCs are not thresholded, the correlation is higher but there seems to be significant variation in the optimum scale required for the SDK model; as shown in (c) and (d). This experiment motivated us to investigate diffusion at multiple scales, resulting in the current proposal of a multi-scale diffusion model. We thank the authors for kindly providing their code which was used for reproducing the results with SDK model.

scale sets, with uniform distribution between $[0, 1]$ (normalized scale space), and trained our model on each of them separately. The generated scale sets had the property that the scales may not be spread equally in the normalized space. Figure S4 plots a histogram of the Pearson correlation coefficients between the test subjects whose mean correlation is plotted in the histogram. The histogram suggests that not only should the scales span the entire space, but they also should be evenly spread in the space. Further it also suggests that the number of scales (16) may be more important than the particular scale values.

1.4 Thresholded SC

To keep edges more than T% of the maximum edge weight, we defined $0 \leq \epsilon \leq 1$ as the factor for thresholding. Based on the mean SC, we selected 6 values for ϵ which are (0.0960, 0.0657, 0.0354, 0.0152, 0.0051, 0.0000) and applied them to get the thresholded empirical SCs. These ϵ's correspond to the data points for 6-T values shown in Figure 4 of the main manuscript. We only selected elements of SC above ϵ times the maximum

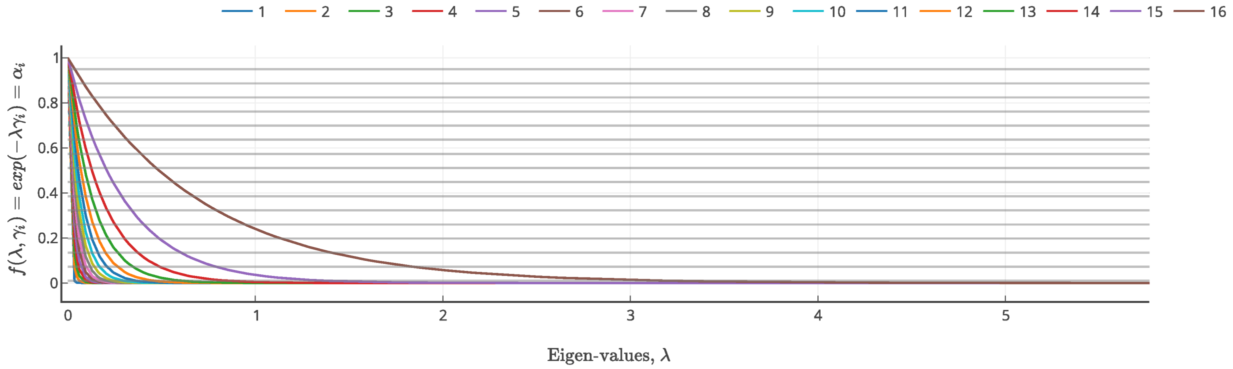

Figure S2: **Procedure for selection of scales**. The procedure for choosing scales from the fixed normalized exponential values and a chosen eigenvalue λ of $\mathbf{L}$. Laplacian matrix $\mathbf{L}$ of each SC is eigen decomposed whose 2nd eigenvalue is used to determine subject-specific scale set from the normalized values, kept the same for all subjects. Each exponential curve is a function of the scale (γ) and represents the contribution of every eigen-component of the Laplacian matrix. Grey horizontal lines denote the fixed normalized scale values. If a larger eigen-value is chosen, then all the large scales will be ignored. Hence, the closer the eigen-value towards 0, the better is the scale variation. Hence, justifying the choice of second eigen-value. Intersection points of all the grey lines on the vertical line at second eigen-value allow only one exponential curve to intersect. The scales determining the exponential curves are the subject-specific scales.

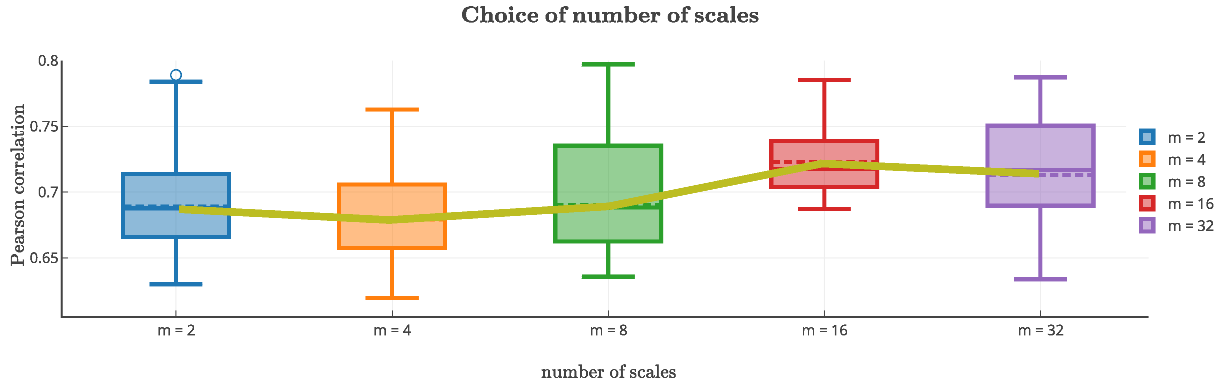

Figure S3: **Choice of number of scales,** m**.** Shown is the MKL model's performance on test subjects. Each bar-plot corresponds to a number_of_scales, m. The thick line inside each box and the dotted line are the median the mean correlation values respectively. As observed, performance increases from $m = 4$ to $m = 16$ and then slightly goes down. For $m = 16$, test performance has the lowest inter-subject variance and highest mean correlation, hence naturally becoming a choice in the design of the model.

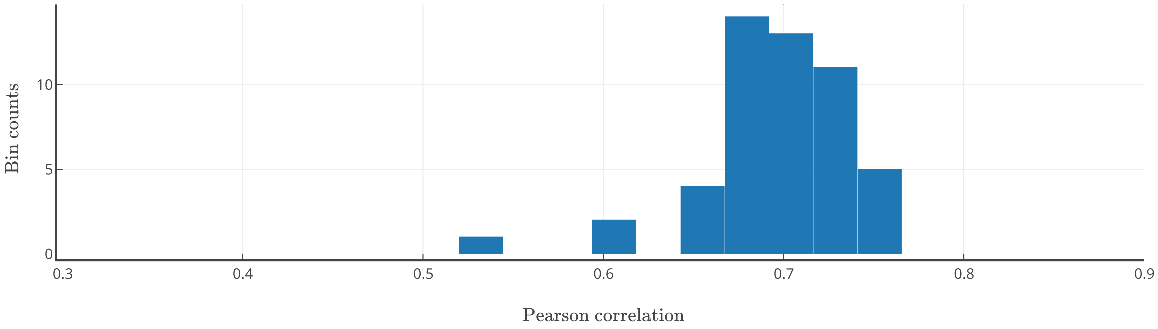

Figure S4: **Procedure for selection of scale configuration**. Behavior of the model is studied by varying scale sets, m being fixed at 16. Each scale set was uniformly sampled from the domain $[0, 1]$. Each scale set consists of m instances of normalized scales. The MKL model was trained and tested on the same split separately for each scale configuration. Histogram shows the mean performance of the models. Eigenvalue of Laplacian $\mathbf{L}$ at second index was used to determine equivalent subject-specific scales. Figure suggests that number of scales is more important than particular scale values. We have shown performance by keeping the normalized scales at uniform intervals.

value of SC. This mask was applied on SC to generate the set of thresholded SCs. Equation 3 describes the pro tess succinctly in the form of a MATLAB® pseudocode statement.

$$\text{SC} \leftarrow (\text{SC} > \epsilon \max(\text{SC})) \odot \text{SC}, \tag{3}$$

where $\odot$ is element-wise product.

1.5 Procedure for perturbation of input

We generated random SC matrices respecting the power law distribution of the connectivities of the ROIs. These are the following steps:

1. We assume that power law distribution takes the form $p_K(k) = k^{1/\alpha+1}$. k being the edge value. We chose α as 2.

2. We generated a random matrix using uniform distribution and applied this function element-wise on the random matrix.

3. Addition of the transpose to itself followed by division with the maximum matrix element provides us with a random SC.

4. We repeated this procedure for all the subjects 250 times.

2 Support for MKL model

This section describes results that boost confidence in the performance of the proposed model. Figure S7 shows scatter plots between mean empirical and predicted FCs for all the models. Evidently, R^2 highlights that variance in prediction of FC in the MKL model is much lesser than that in the other models.

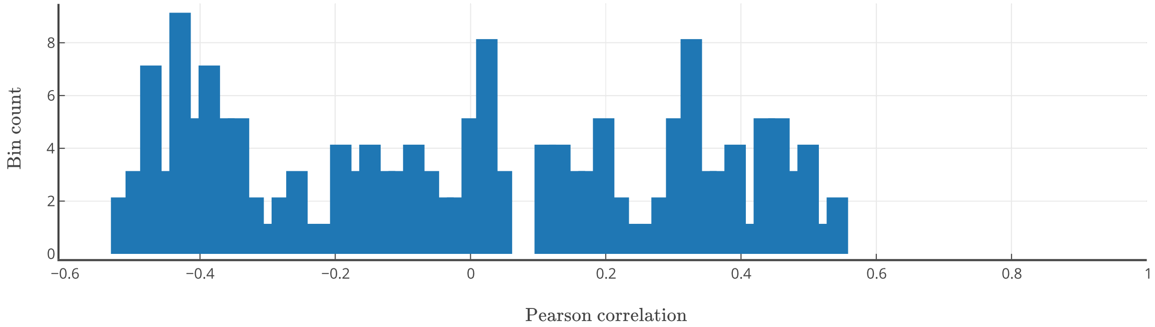

Figure S5: **Input perturbation while training MKL model**. The 250 perturbed sets of SCs were used train independent MKL models. These trained models were used for testing with the correct test SC-FC pairs. Histogram shows the mean performances across all the sets. As observed, most of the mean correlations are around -0.2.

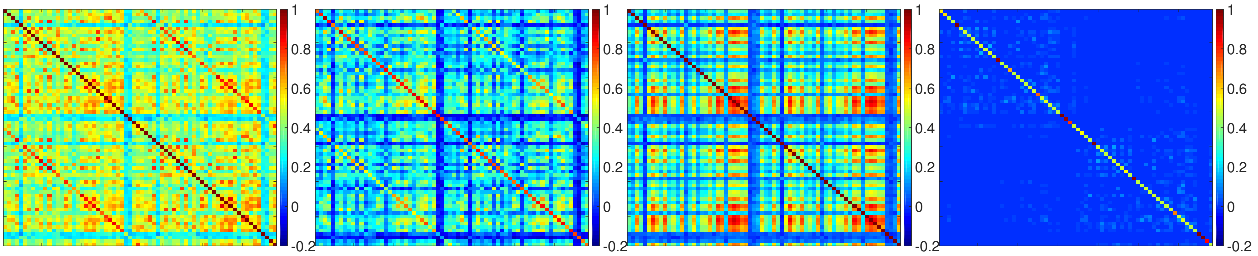

(a) Empirical

(b) MKL, 0.71

(c) DMF, 0.52

(d) SDK, 0.37

Figure S6: **Comparison of Group-mean Connectivity Matrices on Test Dataset**. (a) Mean Empirical FC. (b-d) Mean matrices of the test-subject-wise predicted FCs by the MKL model, DMF model and SDK model, respectively. Pearson correlation values between empirical and predicted FCs are mentioned in the captions for each sub-figures. MKL model captures the finer details present in the empirical FC as compared to the other models. SDK model tries to fit only one scale without inter-regional interactions. Whereas SDK model is only able to partially predict FC, the DMF model fits FC globally well. Compared to the MKL model, DMF model seems to over-predict the connections.

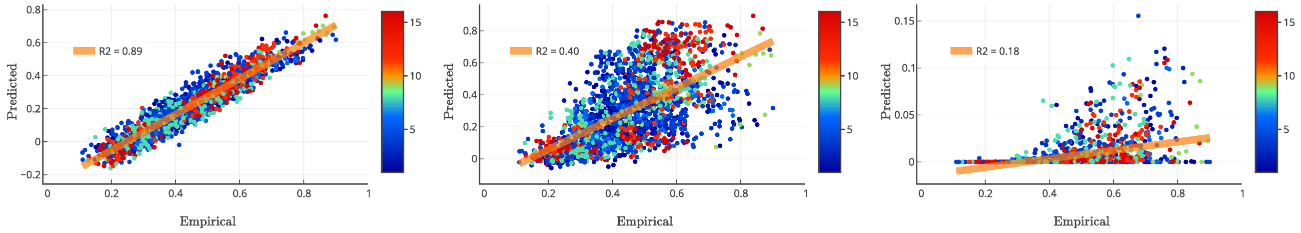

(a) MKL (b) DMF (c) SDK

Figure S7: **Comparison of Scatter Plots**. Scatter plots for mean FCs of the test-subject-wise empirical vs predicted FCs for (a) MKL; (b) DMF; and (c) SDK models. Only the non-diagonal lower triangular matrix from all the FCs has been taken to generate the plots. Each connection was identified as belonging to one of the four different lobes represented by their color codes; resulting in 16 colors representing 4×4 inter-hemispheric connections. Higher R^2 value for the MKL model suggests tighter bound on the element-wise error while predicting FC. Even though prediction of DMF model appears to be closer to that of MKL model, predicted elements are much more scattered for the DMF model.

3 Graph, Laplacian, and Diffusion Kernel

A graph defines an underlying non-euclidean space for a set of elements, possibly in euclidean space, through some pairwise similarity measure between them. These elements are mathematically abstracted as vertices, and the pairwise relationships as edges [2]. Mathematically, a graph is defined as an ordered set $G = (V, E)$, where $V = (v_1, \cdots, v_n)$ is the set of vertices and $E = \{(v_i, v_j)|(v_j \sim v_i) = w_{i,j} \geq 0\}$ is the set of edges. A graph can be represented by the square adjacency matrix $\mathbf{W}_{n\times n}$ whose $(i,j)^{th}$ entry $w_{i,j}$ is the similarity between vertices v_i and v_j. The theory that analyses such underlying non-euclidean topology through eigenvalue-eigenvector spectrum of graph is called spectral graph theory [[3]]. An important matrix capturing properties of the graph is its Laplacian matrix $\mathbf{L}_{n\times n}$. $\mathbf{L}_{n\times n}$ can be understood as the gram matrix of the incidence matrix of the graph. It is defined as $\mathbf{L} = \mathbf{D} - \mathbf{W}$, where $\mathbf{D}$ is the diagonal degree matrix.

For our study, we consider structural connectivity matrix (SC) as the underlying graph adjacency matrix and signal $\mathbf{u}(v_i, t)$ varying both in the underlying space, at every vertex v_i, and at time t. Laplacian matrix takes the form as follows and is eigen-decomposable, considering $\mathbf{\Lambda}_{n\times n}$ to be the diagonal eigenvalue matrix and $\mathbf{\Psi}_{n\times n}$ to be the eigen vector matrix, respectively:

$$\mathbf{L} = \mathrm{D} - \mathrm{SC} = \mathbf{\Psi}\mathbf{\Lambda}\mathbf{\Psi}^\top. \tag{4}$$

Laplacian matrix can be understood as an operator through which other kernels operating on signals on graphs can be derived. A kernel defined using Laplacian matrix is diffusion kernel $\mathbf{H}_i$, also called heat kernel, which is a solution of the heat equation on graphs. This diffusion kernel acts as a spatial diffusion operator derived in the supplementary material of [[4]]. Diffusion kernel is defined on a graph uniquely at a particular scale γ_i:

$$\mathbf{H}_i = e^{-\mathbf{L}\gamma_i} = \mathbf{\Psi} e^{-\mathbf{\Lambda}\gamma_i}\mathbf{\Psi}^\top, \tag{5}$$

second equality coming from Taylor approximation of the exponential matrix. Kondor and Lafferty [[5]] presented diffusion kernels as matrices resembling covariance of a stochastic process on a graph. This motivates us to interpret functional connectivity in terms of diffusion kernels. Another interpretation of graph Laplacian is connected to Fourier transform on non-euclidean spaces. For a periodic 1-D signal, Fourier basis are eigen-functions of the Laplace operator. Extending this idea Shuman et al [[6]] proposes Fourier transform on graphs using eigenvectors of graph as Fourier basis. Thus, any signal $\mathbf{u}$ on a graph can be represented in terms of its components $\hat{\mathbf{u}}$ on the supported harmonics on the graph:

$$\mathbf{u} = \mathbf{\Psi}\hat{\mathbf{u}} \tag{6}$$

$$\hat{\mathbf{u}} = \mathbf{\Psi}^\top\mathbf{u} \tag{7}$$

4 Identifying Inter-group Variations

This section describes an application using $\mathbf{\Pi}$. We used $\mathbf{\Pi}$ to learn the functional patterns in two age groups, young and old. Before learning $\mathbf{\Pi}$'s for both age groups, we sought to validate the requirement of multiscale model instead of a single-scale one. We conducted these experiments on the same dataset used in this paper: The *Berlin data set* comprises young-age group covering ages less than 30 years and old-age group comprising more than 50 years. Figure S8 shows Pearson correlation curves for subjects categorized by their age groups. Albeit data tends to satisfy single-scale hypothesis, the optimum scale seems to be same for both the age-groups. However, the scales and the actual $\mathbf{\Pi}$'s for the two age groups are significantly different as can be inferred from Figure S9.

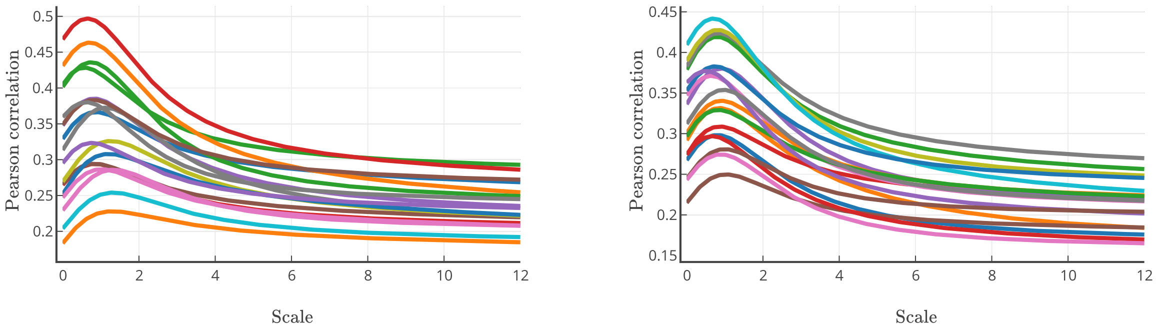

(a) Young age group

(b) Old age group

Figure S8: **Inter-group variations**. Pearson Correlation curves for two age groups: (a) Young age group; (b) Old age group. Looking at the figure, is difficult to comment on the age group of the subject.

Figure S9 shows the differences $\mathbf{\Pi}$ captures between the two age groups. Clearly, elements of $\mathbf{\Pi}$ span different ranges at global scales and these ranges come closer near local scales of diffusion (that is, for higher indices in Figure S9). Moreover, different regions participate in diffusion process in the young and old age groups. We call $\mathbf{\Pi}$ learned from young age group as $\mathbf{\Pi}_{young}$ and similarly that from old group as $\mathbf{\Pi}_{old}$. To further highlight the differences, we used $\mathbf{\Pi}_{young}$ and $\mathbf{\Pi}_{old}$ for predicting subject specific FC's. Figure S10 captures the predictive power of $\mathbf{\Pi}$'s learned separately for the two age groups. We compared subject-wise Pearson correlations with the native and other age group's $\mathbf{\Pi}$. The two plots clearly show that native $\mathbf{\Pi}$'s predict subject-specific FCs better than other group's $\mathbf{\Pi}$'s.

5 Model inversion

This section describes a procedure to recover the structural connectivity (SC) from the functional connectivity (FC) data, given $\boldsymbol{\pi}$'s. The procedure is very simple and described in the following steps.

1. Find the set of diffusion kernels for subject s by solving the linear system of equations.

$$[\boldsymbol{\pi}^1, \cdots, \boldsymbol{\pi}^m] \begin{bmatrix} \mathbf{H}_1^s \\ \vdots \\ \mathbf{H}_m^s \end{bmatrix} = \mathrm{FC}^s. \tag{8}$$

2. As the set of exponential values is known, and we choose second index of eigenvalues for finding the scale set; when each diffusion kernel is eigen-decomposed, the second eigenvalue of these $\mathbf{H}_i$'s should be

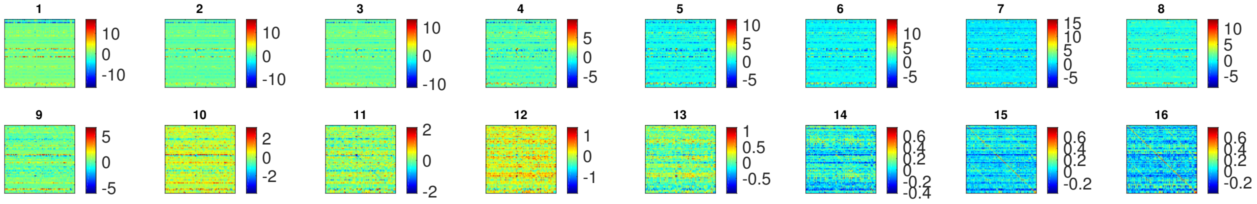

(a) Young age group

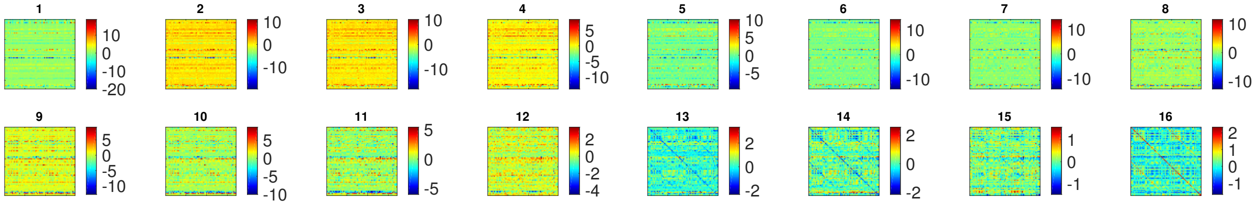

(b) Old age group

Figure S9: **Age-group specific $\mathbf{\Pi}$'s**. Different age groups learn significantly different $\mathbf{\Pi}$'s. Clearly, the set of regions influencing other regions are different in both the age groups. Moreover, ranges of $\mathbf{\Pi}$'s even for individual scale-components are also different. The numbers in the title of each sub figure index the scale-components. Thus, $\mathbf{\Pi}$ can be considered as a viable model for classification.

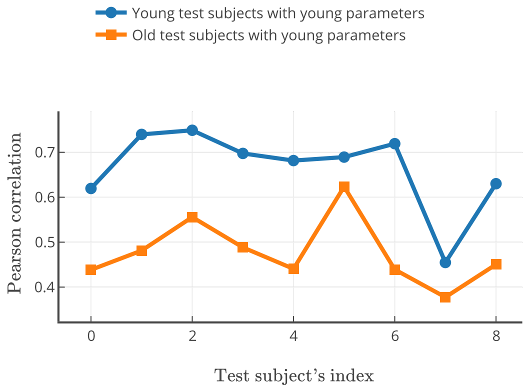

(a) Young age group.

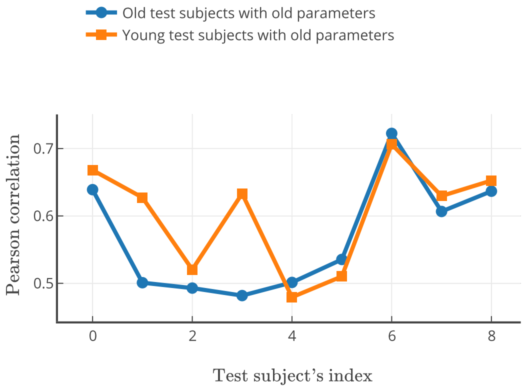

(b) Old age group.

Figure S10: **Comparing the performances of the learned $\mathbf{\Pi}$'s on both age-groups**. Performance of the young age group was tested on both $\mathbf{\Pi}_{young}$ and $\mathbf{\Pi}_{old}$. Similarly old age group's performance was also tested with both the $\mathbf{\Pi}$'s. As seen, $\mathbf{\Pi}$'s capture behavior of both groups and are able to distinguish between them.

1.

$$\mathbf{H}_i^s = e^{-\mathbf{L}^s \gamma_i} = \mathbf{\Psi}^s e^{-\mathbf{\Lambda}^s \gamma_i} \mathbf{\Psi}^{s\top} \tag{9}$$

$$\mathbf{\Lambda}^s = \begin{bmatrix} \lambda_1^s/\lambda_2^s & & & \\ & 1 & & \\ & & \ddots & \\ & & & \lambda_1^s/\lambda_2^s. \end{bmatrix} \tag{10}$$

3. Laplacian $\mathbf{L}^s$ can be obtained from the eigenvector-eigenvalue matrices obtained from the previous step.

$$\mathbf{L}^s = \mathbf{\Psi}^s \mathbf{\Lambda}^s \mathbf{\Psi}^{s\top} = \mathrm{D}^s - \mathrm{SC}^s.$$

4. Removing the diagonal entries of $\mathbf{L}^s$ and negating the remaining values, we obtain SC^s.

Note that this method has some numerical inconsistencies which need to be resolved before it can be deployed.

6 Data Description and pre-processing

All participants were asked to give diagnostic psychiatric interviews in order to acquire comprehensive phenotypic information for the purpose of exploring brain/behavior relationship. We used the data comprising of 47 healthy participants (age: 18-80; mean: 41.55 years; 19 males) scanned at the Berlin Centre for Advanced Imaging, Charité University, Berlin, Germany. All participants gave written informed consent and the study was performed under compliance of laws and guidelines approved by the ethics committee of Charité University, Berlin. Participants did not show any sign of age-related neurodegenerative diseases under clinical testing procedures at the time of imaging. Details of preprocessing are mentioned in Vattikonda et al. [7].

6.1 Empirical DTI data and tractography

For first dataset, images were taken with the following parameters: acquisition time=13:32, TR=10000ms, TE=91ms. Images have 64 diffusion directions with b_values=1000s/mm2. DTI data was corrected for motion and eddy current, skull stripped, and fiber assignment was done using (FACT) algorithm [?]. Fractional anisotropy map was registered to MNI152 template. voxels were parcellated into 188 regions using Craddock's spectral clustering parcellations [?]. We downloaded data from the website whose details are in [8]. The empirical SC matrix of second dataset is generated by using an automated pipeline [9] for reconstruction of fiber tracks from T1 structural MR images and Diffusion-weighted images (DWI). Diffusion-Tensor-Imaging (TR 7500 ms, TE 86 ms, 61 transversal slices (2.0 mm), voxel size 2.3 × 2.3 × 2.3 mm, FoV 220 mm, 96 × 96 matrix) and GRE field mapping (TR 674 ms, TE1 5.09 ms, TE2 7.55 ms, 61 transversal slices (2.3 mm), were measured directly after the anatomical scans. The images obtained from these scans are used as input to the reconstruction pipeline to generate the SC matrix for each subject (Please refer to [9] for a detailed outline of the pipeline for generating SC matrix). In this pipeline, high resolution T1 anatomical images are used to create segmentation and parcellation of cortical and subcortical gray matter. For each subject, binary white matter(WM) masks were used to restrict tracking to WM voxels. dw-MRI data are pre-processed using FREESURFER after extracting gradient vectors and values (known as b-table) using MRTrix. Upon extraction of gradient vectors and values using MRTrix, dw-MRI data are pre-processed using FREESURFER's Using the registration rule created by FREESURFER's function dt-recon we transform the high-resolution mask volumes from the anatomical space to the subject's diffusion space, which will be used for fiber tracking. The cortical and subcortical parcellations contained in aparc+aseg.nii are resampled into diffusion space, one time using the original 1 mm isotropic voxel size (for subvoxel seeding) and one time matching that of our dw-MRI data, i.e., 2.3 mm isotropic voxel size. Based on that, a fractional anisotropy (FA) and an eigenvector map are computed and masked by the binary WM mask created previously. In order to improve existing methods for capacities estimation the approach makes use of several assumptions with regard to seed-ROI selection, tracking and aggregation of generated tracks [9]. Upon tractography the pipeline aggregates generated tracks to structural connectome. The normalized weighted distinct connection counts used here contain only distinct connections between each pair of regions yielding a symmetric matrix.

6.2 Imaging Protocol and Functional Connectivity Matrices

Resting-state fMRI for first dataset was performed on a Siemens Trio 3T with acquisition time = 10:55, TR = 2500 ms, TE = 30 ms, on 38 slices with a voxel size = 3 mm3. fMRI data was slice time corrected, linearly registered, skull stripped, and spatially smoothed. All samples were registered to MNI152 atlas template. Residual BOLD data analyzed with the method elaborated in [?]. Time series of all pairwise ROIs were correlated to calculate FC matrix. Functional MRI and T1-weighted scans for second dataset were acquired using using a 3 Tesla Siemens Trim Trio MR scanner and a 12-channel Siemens head coil. BOLD time-series were acquired at TR 1940 ms lasting 22 minutes (TE 30 ms, FA 78°, 32 transversal slices (3 mm), voxel size 3 x 3 x 3 mm, FoV 192 mm). For functional imaging, subjects were asked to keep awake and keep their eyes closed—no other controlled task had to be performed. In addition a localizer, DTI and T2 sequence were recorded for each subject. Each scan session started a localizer sequence (TR 20 ms, TE 5 ms, 3 slices (8 mm), voxel size $1.9 \times 1.5 \times 8.0$ mm, FA 40°, FoV 280 mm, 192×192 matrix). For each participant anatomical T1-weighted scans (TR 1900 ms, TE 2.25 ms, 192 sagittal slices (1.0 mm), voxel size $1 \times 1 \times 1$ mm, FA 9°, FoV 256 mm, 256×256 matrix) as well as T2-weighted scans (TR 2640 ms, TE1 11 ms, TE2 89 ms, 48 slices (3.0 mm), voxel size $0.9 \times 0.9 \times 3$ mm, FA 150, FoV 220 mm, 256×256 matrix) were acquired. The Virtual Brain pipeline was used for the preprocessing of the data. Further details regarding the preprocessing steps and image acquisition parameters can be found in [9].

6.3 FMRI connectivity

In order to generate the functional connectivity (FC) matrices, raw fMRI DICOM files are first converted into a single 4D Nifti image file. After this step, FSL's FEAT pipeline is used to perform the following operations: deleting the first five images of the series to exclude possible saturation effects in the images, high-pass temporal filtering (100 seconds high-pass filter), motion correction, brain extraction and a 6 DOF linear registration to the MNI space. Functional data is registered to the subject's T1-weighted images and parcellated according to FREESURFER's cortical segmentation. By inverting the mapping rule found by registration, anatomical segmentations are mapped onto the functional space. Finally, average BOLD signal time series for each region are generated by computing the mean over all voxel time-series of each region. From the region wise aggregated BOLD data, FC matrices are computed within MATLAB using pairwise mutual information (on z-transformed data), and Pearson's linear correlation coefficient as FC metrics. Any pre-processing technique, which implies a normalization of data must be avoided when using our analytic operation, for this reason it is important to stress that we did not perform global signal regression on data. Global regression, in fact, changes the distribution of the eigenvalues of the FC and, in particular, shifts the correlations towards negative values. In resting- state BOLD data, this means that zero and negative correlations are introduced. While the debate on the meaning of these negative correlations and on the appropriateness of the use of global regression is open, this procedure must absolutely be avoided when using the analytical operation here presented as the introduction of zero eigenvalues leads to the impossibility of inverting FC to obtain SC. This, not only causes the loss of some information, but it is also based on the assumption that the distribution of the positive eigenvalues is unaffected by global regression, which is not the case as all the eigenvalues become more negative. The major steps involved in generating rs-FC matrix are brain extraction, motion correction, six-degrees of freedom (DOF) linear registration to the MNI space and high pass temporal filtering. Each participant's functional images were registered to pre-processed T1-anatomical images and parcellated into 68 regions of interest (ROIs) using FREESURFER's Desikan-Killiany atlas [10]. Regional time-series were obtained by considering weighted average from voxel-wise time-series. Subject-specific resting state functional connectivity (rs-FC) matrices were obtained by applying z-transformed pairwise Pearson correlation between each pairs of regional BOLD time-series.

References

[1] F. Abdelnour, H. U. Voss, and A. Raj, "Network diffusion accurately models the relationship between structural and functional brain connectivity networks," *Neuroimage*, vol. 90, pp. 335–347, 2014.

[2] U. V. Luxburg, "A tutorial on spectral clustering," 2007.

[3] F. R. Chung, *Spectral graph theory.* American Mathematical Soc., 1997, vol. 92.

[4] S. Atasoy, I. Donnelly, and J. Pearson, "Human brain networks function in connectome-specific harmonic waves," *Nature communications*, vol. 7, 2016.

[5] R. Kondor and J. Lafferty, "Diffusion kernels on graphs and other discrete structures," in *Proc. Int. Conf. on Machine Learning*, 2002, pp. 315–322.

[6] D. I. Shuman, S. K. Narang, P. Frossard, A. Ortega, and P. Vandergheynst, "The emerging field of signal processing on graphs: Extending high-dimensional data analysis to networks and other irregular domains," *IEEE Signal Processing Magazine*, vol. 30, no. 3, pp. 83–98, 2013.

[7] A. Vattikonda, B. R. Surampudi, A. Banerjee, G. Deco, and D. Roy, "Does the regulation of local excitation–inhibition balance aid in recovery of functional connectivity? a computational account," *NeuroImage*, 2016.

[8] J. A. Brown, J. D. Rudie, A. Bandrowski, J. D. Van Horn, and S. Y. Bookheimer, "The ucla multimodal connectivity database: a web-based platform for brain connectivity matrix sharing and analysis," *Frontiers in neuroinformatics*, vol. 6, p. 28, 2012.

[9] M. Schirner, S. Rothmeier, V. K. Jirsa, A. R. McIntosh, and P. Ritter, "An automated pipeline for constructing personalized virtual brains from multimodal neuroimaging data," *Neuroimage*, vol. 117, pp. 343–357, 2015.

[10] R. S. Desikan, F. Ségonne, B. Fischl, B. T. Quinn, B. C. Dickerson, D. Blacker, R. L. Buckner, A. M. Dale, R. P. Maguire, B. T. Hyman *et al.*, "An automated labeling system for subdividing the human cerebral cortex on mri scans into gyral based regions of interest," *Neuroimage*, vol. 31, no. 3, pp. 968–980, 2006.
